# A Meta-Analysis and Genome-Wide Association Study of Platelet Count and Mean Platelet Volume in African Americans

**DOI:** 10.1371/journal.pgen.1002491

**Published:** 2012-03-08

**Authors:** Rehan Qayyum, Beverly M. Snively, Elad Ziv, Michael A. Nalls, Yongmei Liu, Weihong Tang, Lisa R. Yanek, Leslie Lange, Michele K. Evans, Santhi Ganesh, Melissa A. Austin, Guillaume Lettre, Diane M. Becker, Alan B. Zonderman, Andrew B. Singleton, Tamara B. Harris, Emile R. Mohler, Benjamin A. Logsdon, Charles Kooperberg, Aaron R. Folsom, James G. Wilson, Lewis C. Becker, Alexander P. Reiner

**Affiliations:** 1 GeneSTAR Research Program, Division of General Internal Medicine, Johns Hopkins School of Medicine, Baltimore, Maryland, United States of America; 2 Department of Biostatistical Sciences, Wake Forest School of Medicine, Winston-Salem, North Carolina, United States of America; 3 Department of Medicine, University of California San Francisco, San Francisco, California, United States of America; 4 Laboratory of Neurogenetics, National Institute on Aging, National Institutes of Health, Bethesda, Maryland, United States of America; 5 Department of Epidemiology and Prevention, Division of Public Health Sciences, Wake Forest University School of Medicine, Winston-Salem, North Carolina, United States of America; 6 Division of Epidemiology and Community Health, University of Minnesota School of Public Health, Minneapolis, Minnesota, United States of America; 7 Department of Genetics, School of Medicine, The University of North Carolina at Chapel Hill, Chapel Hill, North Carolina, United States of America; 8 Health Disparities Research Section, Clinical Research Branch, National Institute on Aging, National Institutes of Health, Baltimore, Maryland, United States of America; 9 Division of Cardiology, University of Michigan Health System, Ann Arbor, Michigan, United States of America; 10 Department of Epidemiology, University of Washington, Seattle, Washington, United States of America; 11 Division of Public Health Sciences, Fred Hutchinson Cancer Research Center, Seattle, Washington, United States of America; 12 Montreal Heart Institute, Montreal, Canada; 13 Laboratory of Personality and Cognition, National Institute on Aging, National Institutes of Health, Baltimore, Maryland, United States of America; 14 Laboratory for Epidemiology, Demography, and Biometry, National Institute on Aging, National Institutes of Health, Baltimore, Maryland, United States of America; 15 Department of Medicine, University of Pennsylvania School of Medicine, Philadelphia, Pennsylvania, United States of America; 16 Program in Biostatistics and Biomathematics, Division of Public Health Sciences, Fred Hutchinson Cancer Research Center, Seattle, Washington, United States of America; 17 Department of Medicine, University of Mississippi Medical Center, Jackson, Mississippi, United States of America; 18 Department of Epidemiology, University of Washington, Seattle, Washington, United States of America; The University of Queensland, Australia

## Abstract

Several genetic variants associated with platelet count and mean platelet volume (MPV) were recently reported in people of European ancestry. In this meta-analysis of 7 genome-wide association studies (GWAS) enrolling African Americans, our aim was to identify novel genetic variants associated with platelet count and MPV. For all cohorts, GWAS analysis was performed using additive models after adjusting for age, sex, and population stratification. For both platelet phenotypes, meta-analyses were conducted using inverse-variance weighted fixed-effect models. Platelet aggregation assays in whole blood were performed in the participants of the GeneSTAR cohort. Genetic variants in ten independent regions were associated with platelet count (N = 16,388) with p<5×10^−8^ of which 5 have not been associated with platelet count in previous GWAS. The novel genetic variants associated with platelet count were in the following regions (the most significant SNP, closest gene, and p-value): 6p22 (rs12526480, LRRC16A, p = 9.1×10^−9^), 7q11 (rs13236689, CD36, p = 2.8×10^−9^), 10q21 (rs7896518, JMJD1C, p = 2.3×10^−12^), 11q13 (rs477895, BAD, p = 4.9×10^−8^), and 20q13 (rs151361, SLMO2, p = 9.4×10^−9^). Three of these loci (10q21, 11q13, and 20q13) were replicated in European Americans (N = 14,909) and one (11q13) in Hispanic Americans (N = 3,462). For MPV (N = 4,531), genetic variants in 3 regions were significant at p<5×10^−8^, two of which were also associated with platelet count. Previously reported regions that were also significant in this study were 6p21, 6q23, 7q22, 12q24, and 19p13 for platelet count and 7q22, 17q11, and 19p13 for MPV. The most significant SNP in 1 region was also associated with ADP-induced maximal platelet aggregation in whole blood (12q24). Thus through a meta-analysis of GWAS enrolling African Americans, we have identified 5 novel regions associated with platelet count of which 3 were replicated in other ethnic groups. In addition, we also found one region associated with platelet aggregation that may play a potential role in atherothrombosis.

## Author Summary

The majority of the variation in platelet count and mean platelet volume between individuals is heritable. We performed genome-wide association studies in more than 16,000 African American participants from seven population-based cohorts to identify genetic variants that correlate with variation in platelet count and mean platelet volume. We observed statistically significant evidence (p-value<5×10^−8^) that 10 genomic regions were associated with platelet count and 3 were associated with mean platelet volume. Of the regions that were significantly associated, we found 5 novel regions that were not reported previously in other populations. Three of these 5 regions were also associated with platelet count in European Americans and Hispanic Americans. All these regions contain genes that are either known to have or potentially may have a role in determining platelet count and/or mean platelet volume. We further found that one of these regions was also associated with agonist-induced platelet aggregation. Further studies will determine the exact role played by these genomic regions in platelet biology. The knowledge generated by this and other studies will not only help us better understand platelet biology but can also lead us to the discovery of new anti-platelet drugs.

## Introduction

While platelets play a fundamental role in hemostasis, they are also important in the development of atherosclerosis and arterial thrombosis [1]. An elevated platelet count has been associated with adverse clinical outcomes after thrombolysis or coronary intervention in patients presenting with acute myocardial infarction and moderate reductions in platelet count by thrombopoietin inhibition were associated with reduced thrombogenesis in a primate model [2]–[4]. The heritability of variation in platelet count is substantial with estimates ranging from 54% to more than 80% [5]–[8]. In the GeneSTAR study, a cohort included in the current meta-analysis, the heritability of platelet count is 67% [9].

Like platelet count, an elevated mean platelet volume (MPV) is also associated with adverse cardiovascular events and its reported heritability is as high as 73% [8], [10]–[12]. The heritability of MPV in the GeneSTAR cohort was 71% [9]. Recent genome-wide association studies (GWAS) and meta-analyses have identified genetic variants associated with these two platelet traits in Caucasians and a Japanese population [13]–[15]. A recent meta-analysis in the CARe Project, involving genotyping of about 50,000 single nucleotide polymorphisms (SNPs) in 2,100 candidate genes, also reported two genetic variants associated with platelet count in African Americans [16]. The genetic variants reported to date explain only a small fraction of the heritability in platelet count and MPV, providing an opportunity for new studies to discover additional genetic variants of importance [15]. Moreover, African Americans have higher platelet counts than Caucasians and additional genetic variants may contribute to this difference [17]. Because of the different allele frequencies and linkage disequilibrium patterns in populations of European and African ancestry, we anticipated that we might discover new genetic loci associated with platelet count and MPV in an African American population compared to Caucasians [18].

We performed a meta-analysis of 7 GWAS studies that included African-American subjects in the Continental Origins and Genetic Epidemiology Network (COGENT) in order to identify novel genetic variants associated with platelet count and MPV.

## Results

**Figure 1:**
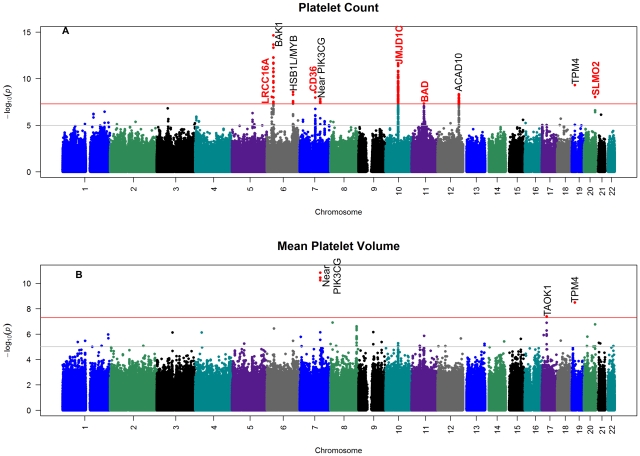
Manhattan plot of the genome-wide association results for meta-analysis. (a) platelet count; (b) mean platelet volume. SNPs are plotted on the x-axis according to their position on each chromosome against the negative log10 of p-values on y-axis. Names of the genes that contain the significant SNPs or are located close to the significant SNPs are indicated on the plot adjacent to the significant SNPs. Names of genes in the novel regions are in red.

**Figure 2:**
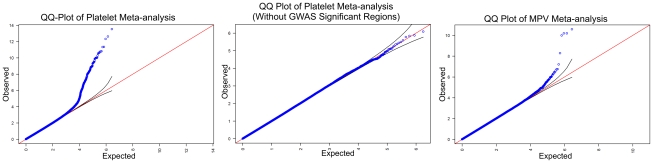
Quantile–quantile (QQ) plots. (a) platelet count meta-analysis with all SNPs included; (b) platelet count meta-analysis after removing 1 million base pairs around the top SNPs from the 10 loci; (c) mean platelet volume meta-analysis. Blue dots are SNPs plotted on the x-axis of expected p-value under the null hypothesis against the observed p-value in the study (p-values are plotted here as negative logarithm 10). The red diagonal line represents the line of unity, the region where expected and observed p-values are the same under the null hypothesis. Black lines above and below the red diagonal line bound 95% confidence intervals. Under the null hypothesis, SNPs should follow the line of unity closely except those SNPs for which the null hypothesis is rejected. SNPs in the QQ plot for the platelet count meta-analysis do not follow the line of unity closely and this appears to be due to large number of significant SNPs in the associated loci. When the chromosomal regions containing these loci are removed, the appearance of QQ plot improves considerably.

We performed a GWAS analysis of platelet count in an African American discovery sample of 16,388 individuals from 7 population-based cohorts (Table 1). The MPV meta-analysis included all subjects from three cohorts and a subset of subjects from two other cohorts (n = 4,531). Following stringent genotyping and imputation quality control procedures (as outlined in the Methods section), over 2.2 million SNPs were available for analysis in each cohort (Table 1). The results of association studies and the genomic-control corrected QQ plot for the combined African-African GWAS analysis for platelet count and MPV are shown in Figure 1 and Figure 2 and study specific QQ plots and genomic inflation factors are reported in Figures S3 and S4 and Table S1. The Jackson Heart Study (JHS) cohort contains a few hundred related individuals. This resulted in a high genomic inflation factor for platelet count and a few other traits, as previously described in Lettre et al [19]. Within the CARe Consortium, Lettre et al have done several analyses involving simulated phenotypes as well as empirical data (lipids, BMI) and have shown that for JHS, genomic control-correction is an appropriate way to control for the small sub-group of related individuals. A list of all genome-wide significant SNPs with regional plots for platelet count and MPV can be found in Tables S2 and S3 and Figure S1. Cohort-specific QQ-plots and association results for index SNPs associated with platelet count or MPV are summarized in Figure S2 and Table S4. Of the 10 loci on 7 chromosomes that reached GWAS threshold (p<5×10^−8^) in the platelet count meta-analysis, five have not been reported in previous platelet count GWAS studies in any population and 8 loci have not been reported previously in African Americans (Figure 1). The MPV meta-analysis identified three loci, each one on different chromosomes; two of these loci were also associated with platelet count at GWAS threshold in the current study (Figure 2). One MPV-associated locus has been reported in African Americans before, and two of these three loci have been associated with MPV in Caucasians in prior studies [15], [16]. A sex-specific meta-analysis did not reveal any heterogeneity for the allelic effect between the two sexes and did not uncover any additional loci. Thus, the sex-specific results are not reported here.

**Table 1:** Characteristics of COGENT African-American meta-analysis cohorts.**

	ARIC	CARDIA	GeneSTAR	HANDLS	Health ABC	JHS	WHI
**Sample size**	2664	943	934	862	898	1992	8095
**Study design** *	unrelated	unrelated	family	unrelated	unrelated	unrelated	unrelated
**Age, years**	53.4 (5.8)	24.4 (3.8)	45.2 (12.6)	48.2 (9.0)	73.4 (2.8)	50.0 (12.1)	61.6 (7.0)
**Female (%)**	63.2	58.7	61.6	56.0	58.8	61.2	100
**SNPs (N)**	2,799,937	2,813,829	3,181,434	3,021,329	3,129,972	2,819,255	2,486,528
**Platelet count (10^9^/L)**	256.4 (65.7)	279.7 (71.4)	265.3 (67.0)	268.7 (76.8)	237.6 (71.6)	256.6 (64.5)	250.3 (60.1)
**MPV** ***	9.3 (0.9) fL	NA	7.9 (0.9) fL	9.2 (1.0) fL	10.9 (1.6) fL	9.2 (0.9) fL	NA

*: All studies are population-based, in addition, JHS has a small group of related individuals.

**: Mean (SD), and units (untransformed) that were used in the regression models for each trait.

***: For MPV, complete cohorts of GeneSTAR, HANDLS, and JHS were included, while only a subset of ARIC (n = 644) and Health ABC (n = 182) were included.

NA = not available; MPV = mean platelet volume.

### Identification of novel loci associated with platelet count and replication in European Americans and Hispanic Americans

The first of the novel loci from platelet count meta-analysis is located on chromosome 6p22. The best SNP (rs12526480; p = 9.1×10^−9^) in this region is located in the intron of the leucine-rich repeat containing 16A gene (*LRRC16A*). The minor allele (G) of rs12526480 was associated with decreased platelet count. Ten additional SNPs in the region had p<10^−6^ (Table 2 and Table S2). The *LRRC16A* gene encodes a protein called ‘capping protein ARP2/3 and myosin-I linker’ (CARMIL), which plays an important role in cell-shape change and motility. Genetic variants in LRRC16A have been previously reported to be associated with serum uric acid levels [20], nephrolithiasis [21] and markers of iron status [22] but there have been no reports of any association with either platelet count or other platelet phenotypes. In the three European American cohorts, rs12526480 was statistically significant in one cohort (p = 0.01) and near nominal significance in the combined meta-analysis (p = 0.06) with an effect size and direction similar to that observed in African Americans. In Hispanic Americans, rs12526480 was not significantly associated with platelet count (Table 3). Given the proximity of the *LRRC16A* gene to the hemochromatosis (*HFE*) gene and the well-known reciprocal relationship between platelet count and iron stores, we additionally assessed the association between rs12526480 and red cell phenotypes in the COGENT African Americans. There was no evidence of association between *LRRC16A* genotype and hemoglobin, hematocrit, red cell count or mean corpuscular volume in the 16,388 African Americans, nor was there any evidence of association between rs12526480 genotype and serum ferritin in 672 African Americans from CARDIA or 2,126 from JHS. Nor did adjustment for red cell phenotype or iron status alter the relationship between platelet count and rs12526480 genotype. Finally, we had uric acid levels available in 943 African Americans from CARDIA; again there was no association with *LRRC16A* genotype (Table S5).

**Table 2:** Novel and validated loci based on genome-wide association with platelet count and mean platelet volume in COGENT (novel loci are in bold).

Locus	Significant SNPs (N)*	Top SNP in region	Position	Candidate gene	Maj/Min Allele	MAF	Effect size (SE)	p-value	Het-P (I^2^)
***PLATELET COUNT***									
**6p22**	1	rs12526480	25641513	LRRC16A	G/T	30.5%	−4.39 (0.76)	9.15×10^−9^	0.62 (0)
6p21	20	rs210134	33648187	BAK1	A/G	28.6%	−6.16 (0.78)	2.32×10^−15^	0.18 (10.6)
6q23	4	rs9494145	135474245	HBS1L, MYB	C/T	7.3%	8.19 (1.38)	2.79×10^−9^	0.99 (0)
**7q11**	2	rs13236689	80073950	CD36	G/T	43.6%	4.18 (0.70)	2.84×10^−9^	0.73 (0)
7q22	4	rs342293	106159455	PIK3CG	G/C	38.6%	−4.05 (0.72)	1.58×10^−8^	0.18 (9)
**10q21**	71	rs7896518	64774506	JMJD1C	G/A	32.4%	5.18 (0.74)	2.26×10^−12^	0.14 (16)
**11q13**	1	rs477895	63805488	BAD	C/T	45.3%	−4.19 (0.77)	4.91×10^−8^	0.17 (11)
12q24	26	rs6490294	110674821	ACAD10	C/A	33.7%	−4.38 (0.75)	4.78×10^−9^	0.71 (0)
19p13	1	rs8109288#	16046559	TPM4	A/G	9.7%	−8.72 (1.40)	5.02×10^−10^	0.35 (0)
**20q13**	1	rs151361	57047397	SLMO2, TUBB1	G/A	25.7%	4.49 (0.78)	9.44×10^−9^	0.04 (40)
***MEAN PLATELET VOLUME***									
7q22	4	rs342296	106160139	PIK3CG	A/G	37.2%	0.16 (0.02)	1.44×10^−11^	0.25 (0)
17q11	1	rs11653144	24699352	TAOK1	C/T	44.2%	−0.13 (0.02)	4.17×10^−8^	0.48 (0)
19p13	1	rs8109288	16046559	TPM4	A/G	8.4%	0.26 (0.04)	3.30×10^−9^	0.84 (0)

*: SNPs in locus reaching GWAS significant threshold (5×10^−8^).

MAF = minor allele frequency; Chr = chromosome; SE = standard error; effect size = age-sex adjusted change in platelet count (10^9^ L) or MPV (fL) per copy of minor allele; Maj = major; Min = minor; Het-P = Cochrane Q p-value to assess heterogeneity.

# excluding cohorts that were included in the previous study^16^ that reported this SNP, the p-value was 8.6×10^−7^.

**Table 3:** Replication of the association of the best SNPs from each novel region with platelet count in three European American cohorts and a Hispanic American cohort.

SNP	Candidate gene	Minor allele	Meta-analysis		ARIC-EA(N = 9274)		WHI-EA (N = 4243)		GeneSTAR-EA (N = 1392)		EA Meta-analysis		WHI-HA (N = 3462)	
			Effect size	p-value	Effect size	p-value	Effect size	p-value	Effect size	p-value	Effect size	p-value	Effect size	p-value
**rs12526480**	LRRC16A	G	−4.39	9.15×10^−9^	−2.29	0.01	0.79	0.54	−2.57	0.32	−1.34	0.06	0.28	0.65
**rs13236689**	CD36	G	4.18	2.84×10^−9^	0.56	0.53	2.4	0.05	1.67	0.51	1.24	0.07		
**rs7896518**	JMJD1C	G	5.18	2.26×10^−12^	5.07	9.65×10^−9^			4.02	0.10	4.95	2.61×10^−9^		
**rs477895**	BAD	C	−4.19	4.91×10^−8^	−3.58	4.48×10^−3^	−2.9	0.088	−0.36	0.92	−3.04	1.71×10^−3^	−3.52	0.04
**rs8109288**	TPM4	A	−8.72	5.02×10^−10^	−18.98	1.49×10^−7^	−11.26	0.03	−8.31	0.33	−15.58	2.60×10^−8^	−12.38	0.02
**rs151361**	SLMO2	G	4.49	9.44×10^−9^	2.71	0.01	2.68	0.066	2.55	0.40	2.69	1.06×10^−3^	0.99	0.55

EA = European Americans; HA = Hispanic Americans.

The second locus is on chromosome 7q11 where two SNPs in intronic regions of the *CD36* gene (rs13236689; p = 2.8×10^−9^ and rs17154155; p = 1.1×10^−8^) reached GWAS significance threshold, while 8 additional SNPs had p<10^−6^. rs13236689 and rs17154155 are in close linkage disequilibrium (r^2^ = 0.90 in the HapMap Yoruban population). After conditioning on rs13236689 in the association analysis, rs17154155 did not remain statistically significant (p = 0.39). Of the three European American cohorts, rs13236689 was statistically significant in the WHI cohort (p = 0.05) but not in the meta-analysis of all three studies (p = 0.07, Table 3). The *CD36* gene encodes a thrombospondin receptor (platelet glycoprotein IV) which is present on the surface of platelets and several other cells [23]. rs17154155 has been reported to be associated with platelet function as well as with platelet expression of CD36 [24], [25].

In the third locus on chromosome 10q21, 71 SNPs reached GWAS threshold and 57 additional SNPs had p<10^−6^. Two non-synonymous common variants of unknown functional significance, rs 10761725 (resulting in serine to threonine substitution) and rs1935 (resulting in glutamate to aspartate substitution), in this region also crossed the GWAS threshold. All 128 SNPs in this region appear to be in strong linkage disequilibrium based on Yoruban HapMap data. The most significant SNP in this region, rs7896518 (p = 2.3×10^−12^), is located in an intron of the jumonji domain containing 1C (*JMJD1C*) gene. SNPs in this region have been reported to be associated with MPV (rs2393967) and with native platelet aggregation in platelet-rich plasma (rs10761741 in Caucasians and rs2893923 in African Americans) but not with platelet count [15], [26]. For rs7896518, data were available from 2 European American cohorts and meta-analysis found a significant association reaching GWAS threshold (p = 2.61×10^−9^) with similar direction of effect size (Table 3).

The fourth novel locus was located on chromosome 11q13. The most significant SNP (rs477895; p = 4.9×10^−8^) was in an intron of the BCL2-associated agonist of cell death (*BAD*) gene, while 23 other SNPs had p<10^−6^. For rs477895, all replication cohorts had effect sizes in a direction similar to African Americans and one European American and the Hispanic cohorts reached statistical significance (p = 4.48×10^−3^ and p = 0.04 respectively). Meta-analysis of the three European American cohorts also found significant association of rs477895 with platelet count (P = 1.71×10^−3^, Table 3). The protein encoded by the *BAD* gene inhibits the activity of the BCL-xL and BCL-2 proteins and thus has a pro-apoptotic effect [27]. Phospholipase C β3 protein encoded by another gene at this locus, *PLCB3*, is also known to be present in platelets and its deficiency results in impaired platelet function in mice [28]. This locus also contains SLC22A11 and SLC22A12, two genes that encode solute carrier proteins and previous GWAS have found association of genetic variants in these genes with serum uric acid levels [20]. Of the two genes, the transcript of SLC22A11 is present in significant amount in platelets as is the transcript for BAD [29]. Interestingly, a SNP about 20 kbp upstream of SLC22A11, rs4930420, almost reached GWAS threshold (p = 9.16×10^−8^, r^2^ with rs477895 = 0.21) and four additional SNPs in complete LD with rs4930420 (r^2^ = 1) had p-values<10^−6^. By examining the actual linkage disequilibrium patterns in this region in COGENT, and by performing conditional regression analysis in more than 8,400 African Americans from the WHI cohort simultaneously adjusting for BAD rs477895 and SLC22A11 rs4930420, we demonstrate that there are likely at least 2 independent platelet count association signals in this region and that the BAD and PLCB3 polymorphisms appear to represent the same association signal (Table S6).

The fifth novel locus was on chromosome 20q13 where one SNP in the *SLMO2* gene exceeded GWAS significance threshold (rs151361; p = 9.4×10^−9^) while 2 other SNPs had p<10^−6^. One of these two SNPs was located in the first intron of *TUBB1* gene (rs6070696; p = 2.5×10^−7^) and was 16.3 kbp downstream of the lead SNP (YRI HapMap r^2^ = 0.6). The *TUBB1* gene encodes a beta1 tubulin, which plays an important role in megakaryopoiesis [30]. All replication cohorts had effect sizes in the direction similar to African Americans for rs151361 but only one European American study reached statistical significance (p = 0.01). The meta-analysis of the three European American replication cohorts also found a statistically significant association between rs151361 and platelet count (p = 1.1×10^−3^, Table 3).

### Validation of previously reported loci for platelet count

In addition to identifying novel loci, we also replicated 5 previously reported loci at GWAS significance threshold and 3 other loci that were highly significant in our study but not at GWAS significance level (Table S7). The strongest signal in our platelet count meta-analysis was from chromosome 6p21 (SNP with the lowest p-value = rs210134; p = 2.3×10^−15^) located in the *BAK1* gene, a locus that has been reported previously in Caucasians, Japanese, and African American populations [13]–[16]. We also found strong associations between platelet count and loci on chromosomes 6q23 (rs9494145; p = 2.8×10^−9^), 7q22 (rs342293; p = 1.6×10^−8^), and 12q24 (rs6490294; p = 4.8×10^−9^), all of which have been previously reported for Caucasians but not for African Americans [15]. Finally, we confirmed the association of a genetic variant rs8109288 (p = 5.0×10^−10^) in the tropomyosin 4 (*TPM4*) gene at chromosome 19p13 that has been previously reported for African Americans in a candidate gene study [16]. In our replication cohorts, rs8109288 was associated with platelet count in meta-analysis of European American cohorts and in Hispanic Americans (p = 2.6×10^−8^ and 0.02 respectively). We were also able to confirm the association of all previously reported SNPs (or a nearby SNP in the same LD block) with platelet count at a p<0.05 (Table S5).

### Identification of loci for MPV

Of the three loci we identified at GWAS significance level for MPV, 2 have been previously reported to be associated with MPV in Caucasians, and one has been reported previously in African Americans. The association which has been previously reported in African Americans was of the A-allele of rs8109288 in *TPM4* with increased MPV (p = 3.3×10^−9^); the same SNP was also associated with platelet count in this study. *TPM4*, a protein with a major role in stabilizing the cellular cytoskeleton, is present in platelets [31]. In the 7q22 region, we found that the SNP with the lowest p-value for MPV (rs342296; p = 1.4×10^−11^) was different from the SNP most associated with platelet count (rs342293; p-value = 5.84×10^−11^) although the two SNPs were only 684 bp apart and are in the same LD block (r^2^ = 0.92 based on HapMap II YRI) [15]. We also replicated a locus associated with MPV on 17q11 (rs11653144; p = 4.2×10^−8^) at GWAS significance threshold [15]. Of the 10 additional previously reported loci for MPV, we found statistically significant associations with 7 of them although these associations did not reach GWAS significance threshold (Table S8). For the loci that we were unable to replicate, we found other nearby SNPs with p<0.05. The direction of effect for all SNPs was not similar to the previously reported study of individuals of European ancestry suggesting that the alleles at the causal loci may be different between the two populations.

### Platelet aggregation studies

Three regions (7q11, 7q22, 10q21) containing four SNPs (rs13236689, rs342296, rs342293, rs7896518) have already been shown to be associated with platelet aggregation [24]–[26], [32]. Therefore, the SNPs with the lowest p-values in each of the remaining 8 regions (Table 4) identified for either platelet count or MPV were examined for their association with platelet aggregation in 832 African-American individuals from the GeneSTAR study. Of the 8 SNPs, 3 were associated with a significant change in agonist-induced platelet aggregation but only one exceeded the Bonferroni-corrected significance threshold of 0.005 (Table 4). The minor allele (C) of rs6490294 in the *ACAD10* gene (12q24) was associated with increased ADP-induced platelet aggregation (p = 0.002). Variants in this region have been previously reported to be associated with coronary artery disease [15]. The minor allele (A) of the 2^nd^ SNP, rs8109288, in the *TPM4* gene, was associated with decreased arachidonic-induced platelet aggregation (p = 0.03) and a trend towards decreased aggregation with ADP (p = 0.09). The minor allele (G) of the 3^rd^ SNP, rs151361, in the *SLMO2* gene, was associated with increased ADP-induced platelet aggregation (p = 0.008). The last 2 SNPs were nominally significant but did not exceed the Bonferroni-corrected significance threshold.

**Table 4:** Association of the top SNP from each locus with agonist-induced platelet aggregation in whole blood.

SNP	Candidate gene (s)	Allele (MAF)	Arachidonic acid		ADP		Collagen	
			P-value	ES	P-value	ES	P-value	ES
rs12526480	LRRC16A	G (0.30)	0.38	0.320	0.13	0.453	0.51	0.252
rs210134	BAK1	A (0.29)	0.42	−0.272	0.94	−0.024	0.46	−0.304
rs9494145	HBS1L, MYB	C (0.07)	0.85	0.117	0.41	0.412	0.87	−0.121
rs477895	BAD	C (0.45)	0.33	−0.333	0.25	−0.338	0.93	0.034
rs6490294	ACAD10	C (0.34)	0.14	0.460	**1.77×10^−3^**	0.827	0.07	0.580
rs11653144	TAOK1	C (0.44)	0.57	−0.181	0.15	−0.414	0.12	−0.659
rs8109288	TPM4	A (0.10)	**0.03**	−1.171	0.09	−0.698	0.35	−0.533
rs151361	SLMO2, TUBB1	G (0.26)	0.54	0.213	**7.95×10^−3^**	0.698	0.11	0.578

Arachidonic acid 0.5 mmol/L, ADP 10 µmol/L, and collagen 5 µg/mL; MAF = minor allele frequency; ES = effect size.

Maximal aggregation was measured in ohms with impedance aggregometry 5 minutes after introducing agonist.

## Discussion

We report the first meta-analysis of GWA studies of platelet count and MPV in a large number of African American participants from 7 population-based cohorts. We have identified 5 novel loci associated with platelet count of which three were replicated in the European American cohorts and one in the Hispanic cohort. None of these new African-American platelet loci have been reported previously in any racial group. In addition, we have confirmed that several loci previously reported in Europeans or Japanese are also associated with these platelet phenotypes in African Americans. We have further shown that 3 of the 8 loci (with one exceeding Bonferroni-corrected threshold), for which there have been no previously known association with platelet aggregation, are also associated with differences in platelet function using a subset of our African American sample.

Interestingly, the 5 novel platelet count loci are intragenic and 4 of these genes are known to have some role in platelet formation or biology. Platelets are small anucleate blood cells that are released from the cytoplasm of much larger bone marrow precursor cells known as megakaryocytes. One of the novel findings is the association of LRRC16A gene with platelet count. The protein encoded by the *LRRC16A* gene, capping protein ARP2/3 *and* myosin-I linker (CARMIL), plays an important role in actin-based cellular processes. Actin filaments are essential for end-amplification of pro-platelet processes during megakaryocyte maturation [33]. CARMIL exposes the barbed ends of actin filaments by binding to and then dislodging the capping protein from the actin filament [34]. Capping proteins are up-regulated during megakaryocyte maturation and LRRC16A is differentially expressed in megakaryocytes compared to other blood cells [35], [36]. The capping protein binding region of the CARMIL protein resides in the later part of the protein (940–1121 amino acid residues), which is a highly conserved region from protozoa to vertebrates. The majority of the residues in this region are critical for the anti-capping protein activity of CARMIL [37]. The rs12526480 genetic variant identified in our study is located in the latter part of the gene and may be in LD with a functional mutation in this conserved region. Any mutation that decreases the ability of CARMIL to dislodge capping protein from the barbed ends of the actin filament may result in abnormal megakaryocyte maturation and decreased platelet formation which is consistent with the direction of effect we observed in our study.

Another novel finding not reported in earlier GWA studies is the association of platelet count with *CD36*, a gene that encodes a receptor present on the surface of platelets, megakaryocytes, and several other cells. CD36 has a wide variety of ligands including thrombospondin [23]. Both *CD36* and thrombospondin genes are up-regulated during megakaryocyte maturation and binding of thrombospondin-I to CD36 inhibits megakaryopoiesis, thus potentially providing a feedback mechanism for control of megakaryopoiesis [34], [36], [38]. The exact mechanism through which activation of CD36 inhibits megakaryopoiesis is unclear but may involve activation of extrinsic apoptotic mechanisms [39].

The most significant SNP associated with platelet count (rs210134 in *BAK1*) in our study is in complete LD with the most significant *BAK1* SNP reported to be associated with platelet count in individuals of European ancestry (rs210135, r^2^ = 1 with rs210134 in HapMap II YRI, p = 2.18×10^−14^ in the current study). While the magnitude of effect is similar, the direction of effect is opposite suggesting that the allele at the causal locus is different in the two ethnic groups. A candidate gene study in African Americans has reported another SNP (rs449242, r2 = 0.81 with rs210134 in HapMap II YRI) in *BAK1* and the direction of effect is similar to our study (Table S5) [16]. In addition to confirming the association of genetic variants in the pro-apoptotic *BAK1* gene with low platelet count, we have identified and replicated a variant in another pro-apoptotic gene, *BAD*, that is associated with low platelet count. The protein encoded by *BAD* acts as a sensor for apoptotic signals upstream of BAK and activates BAK through indirect mechanisms [27]. The identification of these two genes in the intrinsic apoptotic pathway highlights the importance of the apoptotic process in modulating platelet lifespan in the circulation, which is one of the mechanisms that regulate platelet count [40]. Interestingly, this region also contains genetic variants associated with serum uric acid levels [20], however, the mechanism through which uric acid levels may be associated with platelet count remains unclear.

Genetic variants in the *JMJD1C* gene have been previously reported to be associated with MPV in Caucasians but not with platelet count. Conversely, we found several SNPs in this region that reached GWAS significance threshold for association with platelet count but none with MPV and we replicated the lead SNP in European Americans at GWAS threshold. In a GWAS study of platelet aggregation in Caucasians, the minor allele (T) of rs10761741 was associated with an increase in epinephrine-induced platelet aggregation in Caucasians [26]. *JMJD1C* gene is a histone demethylase and appears to be involved in steriodogenesis [41]. In addition to its association with platelet aggregation and MPV, previous GWAS have found genetic variants in this gene to be associated with serum levels of alkaline phosphatase and lipoprotein particle size and content [42]–[44].

In addition to confirming the finding of association of A-allele of rs8109288 in *TPM4* gene with lower platelet count [16] and replicating this finding in European Americans, we also confirmed the association of the A-allele of this SNP with increased MPV and found a nominally significant association with decreased platelet aggregation. *TPM4* gene expression is higher in megakaryocytes than other blood cells or other hematopoietic cells [35], [45]. Tropomyosin proteins play a central role in actin-based cytoskeletal changes and there appears to be biological plausibility for an effect of genetic variants on megakaryocyte maturation and platelet aggregation [46].

The final novel locus in the *SLMO2* gene was also replicated in European Americans but *SLMO2* gene has no known role in megakaryocyte biology. However, the variant is located within 13 kb of the *TUBB1* gene, which is essential in the formation of normal mature platelets. The *TUBB1* gene encodes beta1-tubulin that is exclusively expressed in platelets and megakaryocytes and forms a component of microtubules [30]. Loss of function mutations in *TUBB1* gene have been reported in the literature and result in thrombocytopenia, large platelets, and increased risk of intracranial hemorrhage in men [47], [48]. The G-allele of the rs1513691 variant is associated with increased platelet count, decreased MPV, and increased aggregation, which may point towards a gain in function mutation in this region.

All previously reported loci that were also significantly associated with platelet count or MPV at GWAS threshold in our study have known biological roles in platelet biology. Two of these regions, 6q23 and 12q24, have pleiotropic effects with the 6q23 region associated with several hematological traits [13], [15], [49] and the 12q24 region associated with celiac disease and coronary artery disease [15]. More importantly, we also found that the 12q24 locus was associated with platelet aggregation after Bonferroni adjustment for multiple comparisons and thus may provide a mechanistic explanation of its role in development of coronary artery disease. The GG genotype of the most significant SNP in the 7q22 region, rs342293, is known to be associated with higher *PIK3CG* mRNA levels in platelets [32]. SNPs at this locus are also associated with platelet aggregation, pulse pressure, and carotid artery plaque [26], [50], [51]. *TAOK1* is an important regulator of the mitotic progression and may also play a role in the apoptosis of cells [52], [53].

Our study included over 16,000 participants with platelet count and over 4500 participants with MPV measured and we were able to identify loci that explain between 0.16–0.33% of the variance in platelet count and loci that explain 1–1.5% of the variance of MPV (Table S9). Overall, the loci we identified explain up to 7% of the variance in platelet count and up to 6% of the variance in MPV, assuming that the each of these loci is independent. However, for both platelet count and MPV, the estimated heritability is >50%. Therefore, for each of these traits, the majority of heritability remains unexplained. One of the limitations of GWA studies is the limited power to detect effects caused by genetic variants with frequency <5%. We hypothesize that a significant proportion of the heritability of platelet count and MPV may be explained by variants with frequency <5%. Alternatively, there may be a large number of additional common variants that affect these traits, but have more modest effects.

In conclusion, we have conducted a meta-analysis of GWAS studies of platelet count and MPV in a large African American population and identified novel genetic variants in regions with genes that are likely to have a role in platelet formation. Furthermore, we have replicated 3 of the 5 novel loci in European Americans and one in Hispanic Americans. The novel regions identified may provide a focus for further research in improving our understanding of the biology of megakaryocyte maturation and platelet survival. In addition, we examined the effect of the genetic variants associated with platelet count and MPV on platelet function, and found 3 of these genetic variants to be associated with agonist-induced platelet aggregation of which one crossed Bonferroni-corrected significance threshold. Whether these newly identified genetic variants contribute to the risk of coronary artery disease or myocardial infarction, or to disorders associated with hyper- or hypo-aggregation of platelets, merits further investigation.

## Methods

### Subjects

The 7 studies included in this meta-analysis belonged to COGENT and enrolled 16,388 African American participants. The supplementary text contains a detailed description of each participating COGENT study cohort (Text S1). All participants self-reported their racial category. Additional clinical information was collected by self-report and clinical examination. All participants provided written informed consent as approved by local Human Subjects Committees. Study participants who were pregnant or had a diagnosis of cancer or AIDS at the time of blood count were excluded. We also excluded subjects who were outliers in the analysis of genetic ancestry (as determined by cluster analysis performed using principal component analysis or multi-dimensional scaling) or who had an overall SNP missing rate >10%.

### Platelet count and MPV measurements

Fasting blood samples for complete blood count (CBC) analysis were obtained by venipuncture and collected into tubes containing ethylenediaminetetraacetic acid. Platelet counts and MPV were performed at local laboratories using automated hematology cell counters and standardized quality assurance procedures. Methods used to measure the blood traits analyzed in this study have been described previously for ARIC, CARDIA, JHS, Health ABC, WHI, and GeneSTAR [54]–[58]. Platelet count was reported as 10^9^ cells per liter, and was recorded in all 16,388 study participants. Information on MPV was available in a subset of 4,612 participants from five COGENT study cohorts (ARIC, GeneSTAR, Health ABC, HANDLS, and JHS) and was reported in femto liters (10^−15^ L). All the phenotypes were approximately normally distributed and we did not perform any data transformations.

### Genotype data and quality control

Genotyping was performed within each COGENT cohort using methods described in Text S1. Affymetrix chips were used in the ARIC, CARDIA, JHS, and WHI studies and Illumina chips were used in GeneSTAR, HANDLS, and Health ABC. DNA samples with a genome-wide genotyping success rate <95%, duplicate discordance or sex mismatch between genetic estimates of gender and self-report, SNPs with genotyping failure rate >10%, monomorphic SNPs, SNPs with minor allele frequency (MAF) <1%, and SNPs that mapped to several genomic locations were removed from the analyses. Because African-American populations are recently admixed, we did not filter on Hardy-Weinberg equilibrium p-value. Instead, significantly associated SNPs were later examined for strong deviations from Hardy–Weinberg equilibrium and/or raw genotype data was examined for abnormal clustering. Participants and SNPs passing basic quality control were imputed to >2.2 million SNPs based on HapMap II haplotype data using a 1∶1 mixture of Europeans (CEU) and Africans (YRI) as the reference panel. Details of the genotype imputation procedure are described further under Supplemental Methods. Prior to meta-analyses, SNPs were excluded if imputation quality metrics (equivalent to the squared correlation between proximal imputed and genotyped SNPs) were less than 0.50.

### Platelet aggregation assays

Differences in platelet count may affect platelet function and aggregation [59]. In addition, younger platelets have higher MPV than older platelets and are more reactive [60]. We hypothesized that the genetic variants that determine platelet count and MPV may also affect platelet aggregation. To examine this hypothesis, we used agonist-mediated platelet aggregation assays, which can provide information about the different aspects of platelet aggregation. For these assays, platelet aggregation agonists, such as collagen or ADP, are added to whole blood or platelet-rich plasma and platelet aggregation is measured after a specified amount of time (300 seconds). We performed platelet aggregation assays in the participants of the GeneSTAR cohort. Blood samples were obtained as described above, and platelet aggregation in whole blood was measured as reported previously [57]. Briefly, in vitro whole blood impedance in a Chrono-Log dual-channel lumiaggregometer (Havertown, Pa) was performed after samples were stimulated with arachidonic acid (0.5 mmol/L, intra-assay CV = 24%), collagen (5 µg/mL; intra-assay CV = 9%), or ADP (10 µmol/L; intra-assay CV = 46%). Maximal aggregation within 5 minutes of agonist stimulation was recorded in ohms.

### Data analysis

For all cohorts, genome-wide association (GWAS) analysis was performed using linear regression adjusted for covariates, implemented in either PLINK v1.07, R v2.10, or MACH2QTL v1.08 [61], [62]. Allelic dosage at each SNP was used as the independent variable, adjusted for primary covariates of age, age-squared, sex, and clinic site (if applicable). The first 10 principal components were also incorporated as covariates in the regression models to adjust for population stratification (Text S1). For GeneSTAR, family structure was accounted for in the association tests using linear mixed effect (LME) models implemented in R [63]. Although the JHS has a small number of related individuals, extensive analyses have shown that results were concordant using linear regression or LME, after genomic control [19]. Therefore, results are presented for JHS using linear regression. For imputed genotypes, we used dosage information (i.e. a value between 0.0–2.0 calculated using the probability of each of the three possible genotypes) in the regression model implemented in PLINK or MACH2QTL (for cohorts with unrelated individuals) or the Maximum Likelihood Estimation (MLE) routines (for GeneSTAR).

For both platelet phenotypes, meta-analyses were conducted using inverse-variance weighted fixed-effect models to combine beta coefficients and standard errors from study level regression results for each SNP to derive a combined p-value and effect estimate [64]. Study level results were corrected for genomic inflation factors (λ_GC_) by incorporating study specific λ_GC_ estimates into the scaling of the standard errors (SE) of the regression coefficients by multiplying the SE by the square-root of the genomic inflation factor. The inflation factors for all completed analyses are presented in Table S1. To maintain an overall type 1 error rate of 5%, a threshold of α = 5×10^−8^ was used to declare genome-wide statistical significance. Between-study heterogeneity of results was assessed by using Cochrane's Q statistic and the I^2^ inconsistency metric. Meta-analyses were implemented in the software METAL [64] and were performed independently by two analysts to confirm results. To examine whether there were any differences between males and females, sex-specific GWAS were conducted in each cohort. The results for each SNP were pooled and heterogeneity of allelic effects between females and males was examined using the meta-analysis methods as implemented in GWAMA software [65].

To assess whether the loci previously reported to be associated with the platelet phenotypes in Europeans, Japanese, and African Americans were replicated in the COGENT African-Americans, we examined the meta-analysis results for each index SNP in the regions previously reported, including consistency of direction of effect. If the reported index SNP was not significant at p<0.05 we examined adjacent SNPs and reported the closest SNP with p<0.05 along with its distance from the index SNP.

To examine the association of genotype on platelet aggregation in the GeneSTAR cohort, linear mixed models were used with additive models adjusting for age and sex, and taking into account familial correlation between the individuals.

## Supporting Information

Figure S1 Negative log(10) statistical significance plots of the each local region with 500 kbp on either side of the top SNP significantly associated with platelet count.
(PDF)

Figure S2 Negative log(10) statistical significance plots of the each local region with 500 kbp on either side of the top SNP significantly associated with mean platelet volume.
(PDF)

Figure S3 QQ plots of individual studies for platelet count (PLT).
(PDF)

Figure S4 QQ plots of individual studies for mean platelet volume (MPV).
(PDF)

Table S1 Genomic inflation factors for all GWAS analyses included in the meta-analysis.
(PDF)

Table S2 List of all SNPs with p-values<10^−6^ in the regions that were significant at GWAS threshold in platelet count meta-analysis.
(PDF)

Table S3 List of all SNPs that were significant at p-value<10−6 in regions with at least one SNP with GWAS threshold in mean platelet volume meta-analysis.
(PDF)

Table S4 Individual study results for the top significant SNP from each region.
(PDF)

Table S5 Association analysis of rs12526480 SNP with selected phenotypes.
(PDF)

Table S6 Conditional analysis of the significant SNPs in at the 11q13 locus for platelet count.
(PDF)

Table S7 Association of loci previously reported with platelet count in Caucasians, Japanese, or African American populations (from references 13, 15, and 16).
(PDF)

Table S8 Association of loci previously reported with mean platelet volume in Caucasians (N = 13943)(from reference [15]).
(PDF)

Table S9 Percentage variance in phenotype explained by each SNP for each study.
(PDF)

Text S1 Supporting Methods.
(DOCX)
