# Supplementary material for: A Meta-Analysis and Genome-Wide Association Study of Platelet Count and Mean Platelet Volume in African Americans
Source: PLoS Genet. 2012 Mar 8;8(3):e1002491. doi: 10.1371/journal.pgen.1002491 (PMC3299192; doi:10.1371/journal.pgen.1002491)
Supplement: Figure S1 — Negative log(10) statistical significance plots of the each local region with 500 kbp on either side of the top SNP significantly associated with platelet count. (PDF) [file pgen.1002491.s001.pdf]

Figure S1 (A-J): Negative log(10) statistical significance plots of the each local region with 500 kbp on either side of the top SNP significantly associated with platelet count

Chromosomal position is on x-axis and –log(10) of p-value is on the y-axis. The color of a circle is based on the r^2 from HapMap II data. A gray circle represents a SNP for which r^2 data is not available from HapMap II. These plots were generated using LocusZoom (http://csg.sph.umich.edu/locuszoom/).

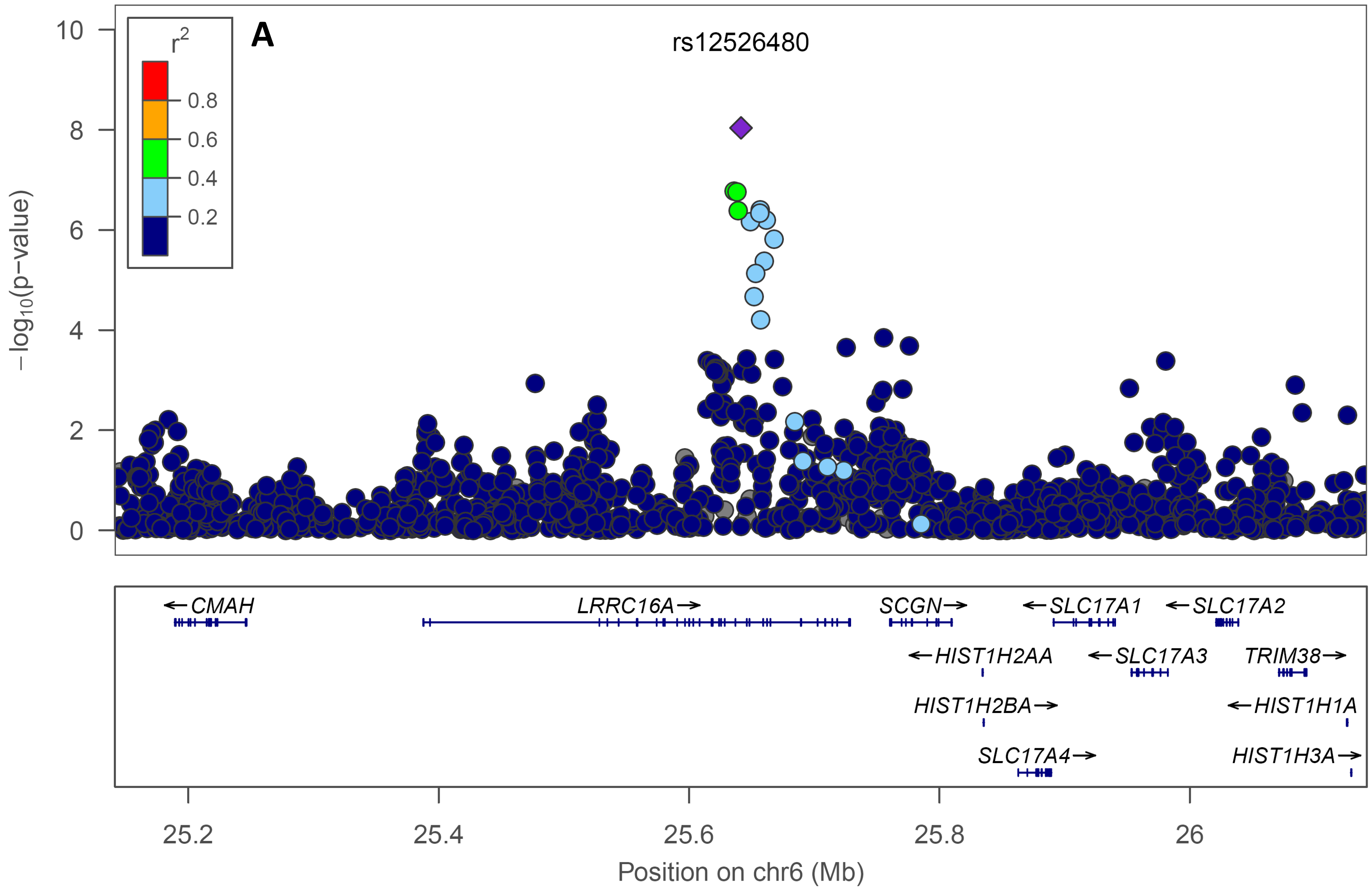

A
rs12526480
r2
0.8
0.6
0.4
0.2
10
8
6
4
2
0
−log10(p−value)
CMAH
LRRC16A
SCGN
SLC17A1
SLC17A2
HIST1H2AA
SLC17A3
TRIM38
HIST1H2BA
HIST1H1A
SLC17A4
HIST1H3A
25.2
25.4
25.6
25.8
26
Position on chr6 (Mb)

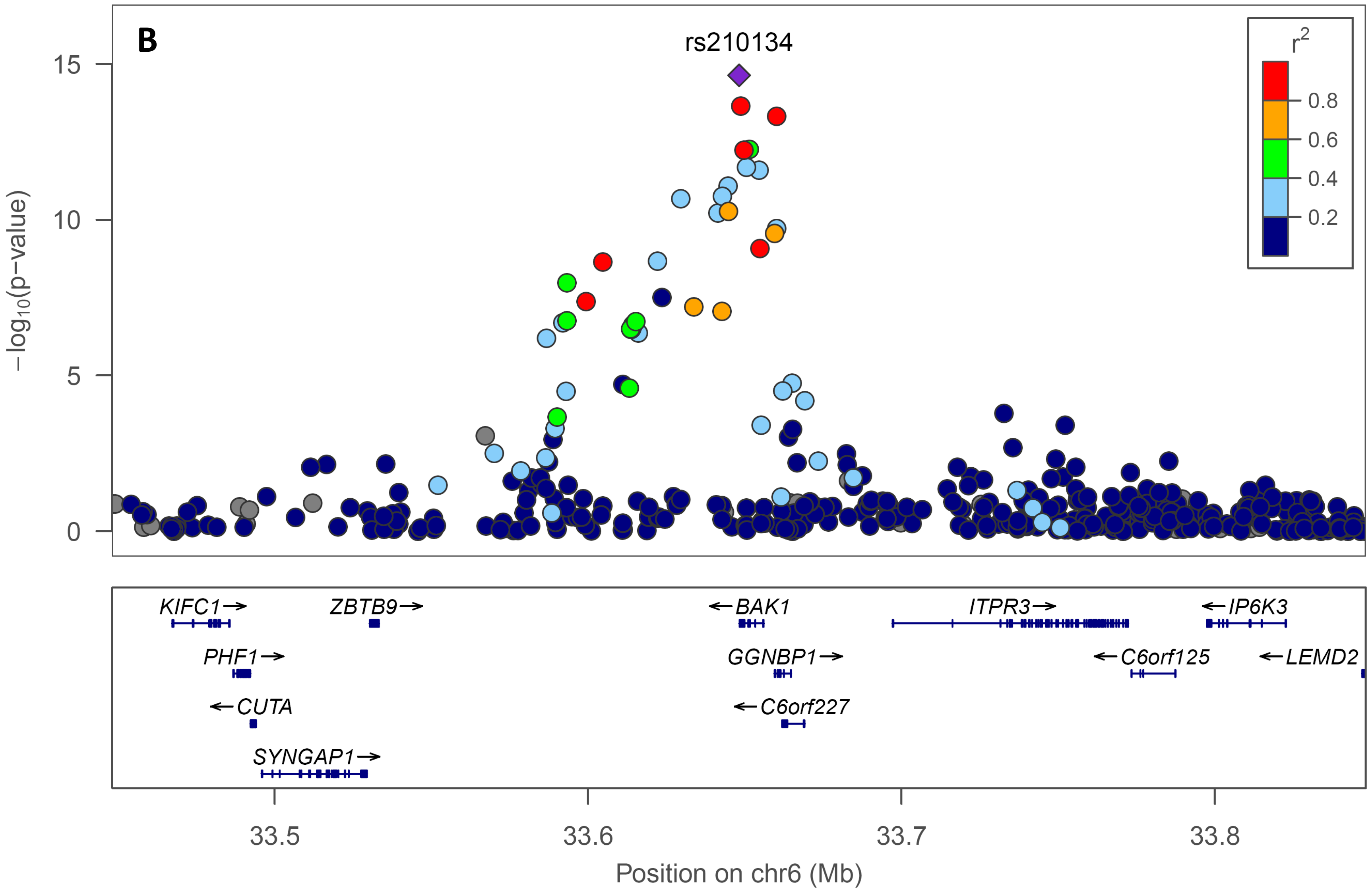
B
rs210134
15
10
5
0
−log10(p−value)
r2
0.8
0.6
0.4
0.2
KIFC1→
ZBTB9→
←BAK1
ITPR3→
←IP6K3
PHF1→
GGNBP1→
←C6orf125
←LEMD2
←CUTA
←C6orf227
SYNGAP1→
33.5
33.6
33.7
33.8
Position on chr6 (Mb)

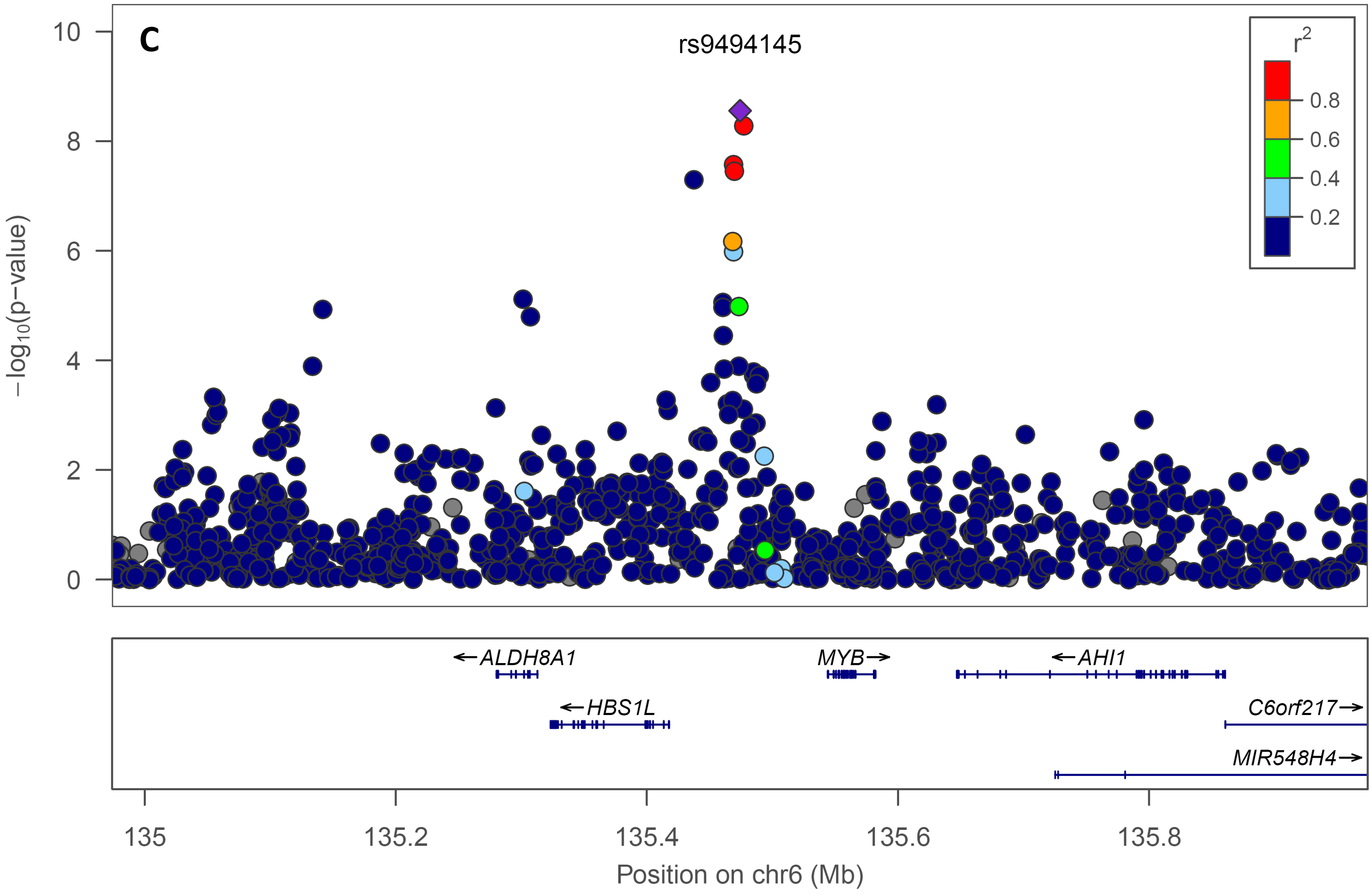
C
rs9494145
r2
0.8
0.6
0.4
0.2
10
8
6
4
2
0
−log10(p−value)
ALDH8A1
HBS1L
MYB
AHI1
C6orf217
MIR548H4
135
135.2
135.4
135.6
135.8
Position on chr6 (Mb)

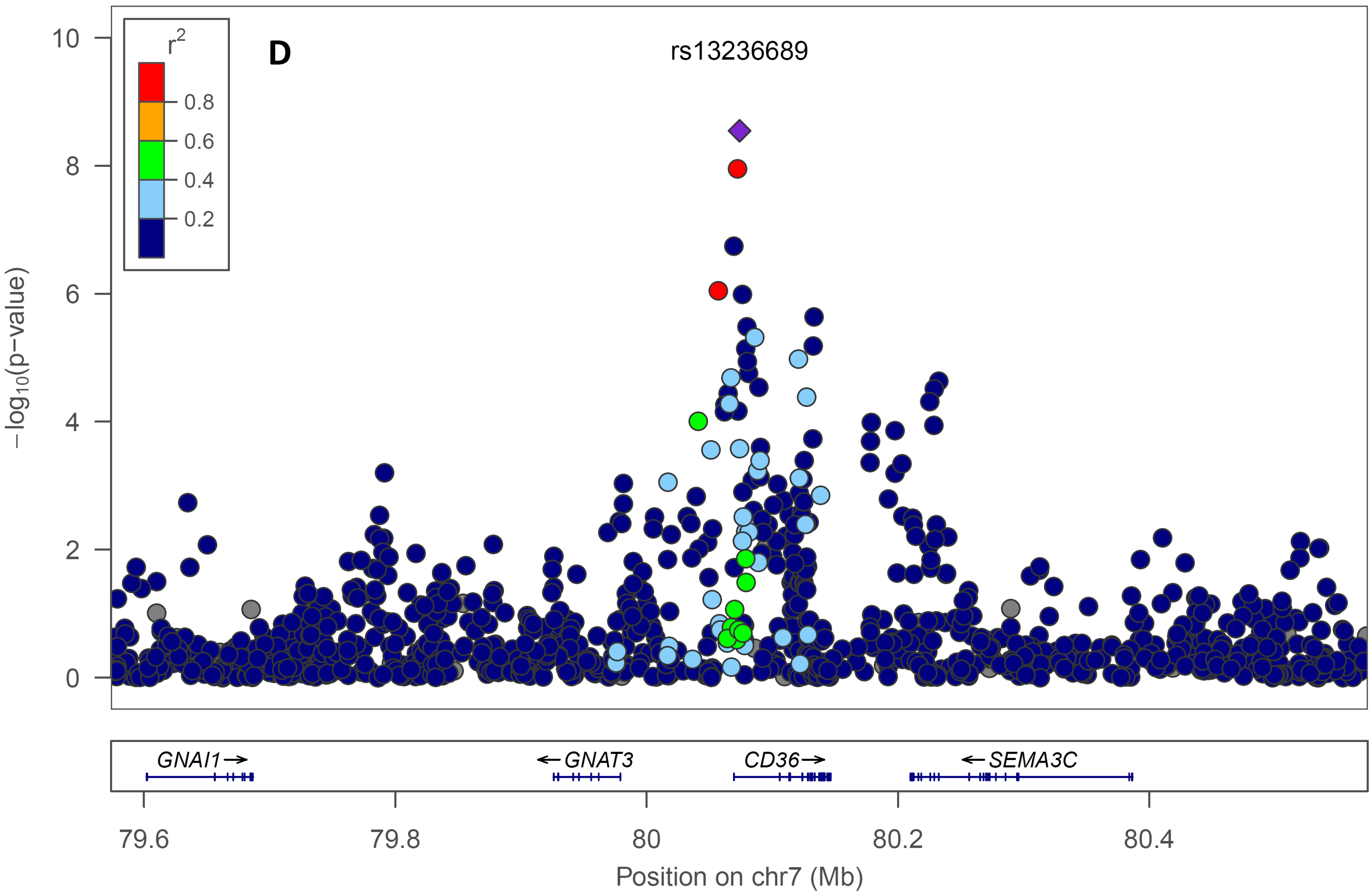

D
rs13236689
r2
0.8
0.6
0.4
0.2
10
8
6
4
2
0
−log10(p−value)
GNAI1→
←GNAT3
CD36→
←SEMA3C
79.6
79.8
80
80.2
80.4
Position on chr7 (Mb)

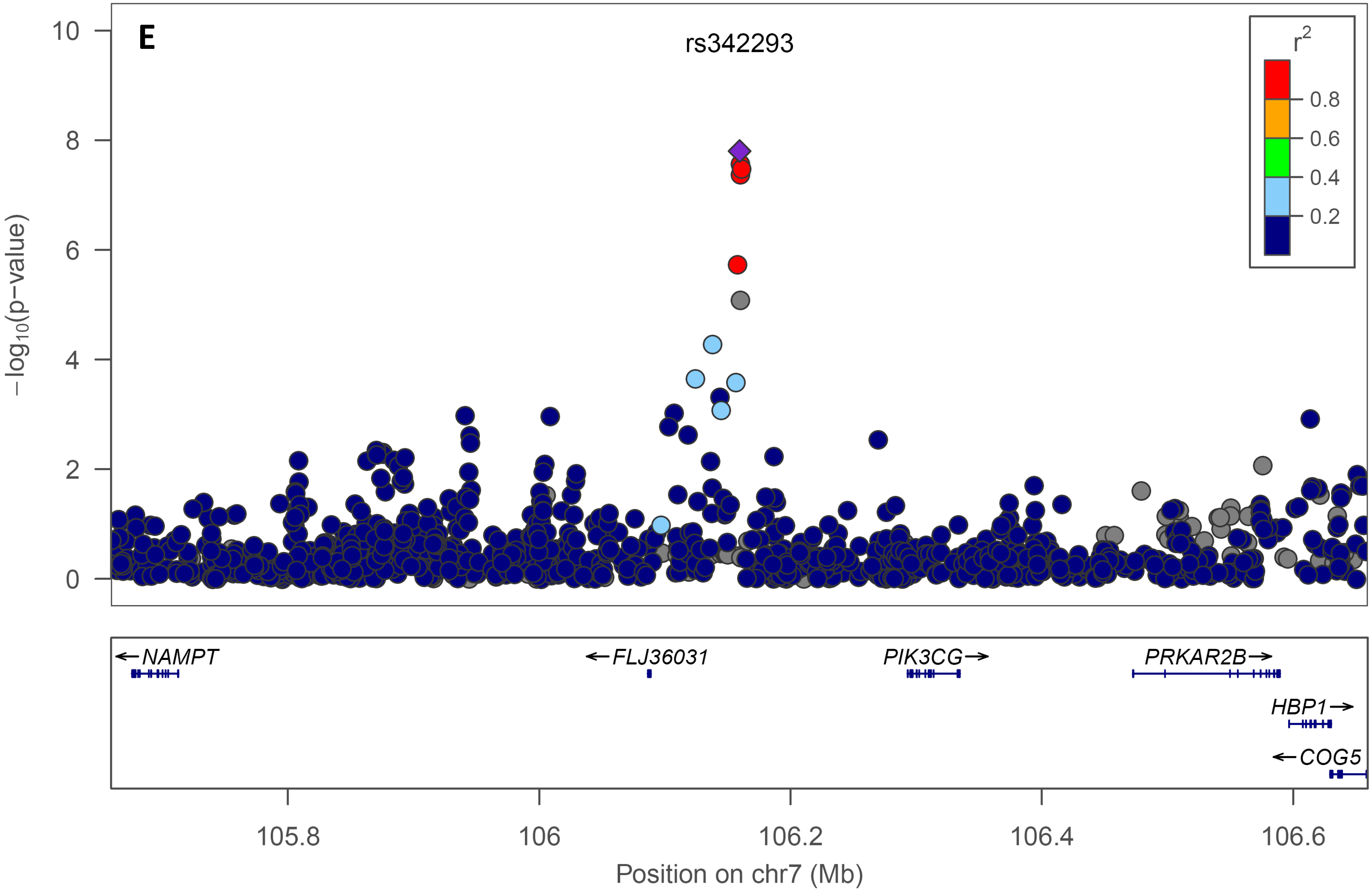

E
rs342293
r2
0.8
0.6
0.4
0.2
−log10(p−value)
10
8
6
4
2
0
NAMPT
FLJ36031
PIK3CG
PRKAR2B
HBP1
COG5
105.8
106
106.2
106.4
106.6
Position on chr7 (Mb)

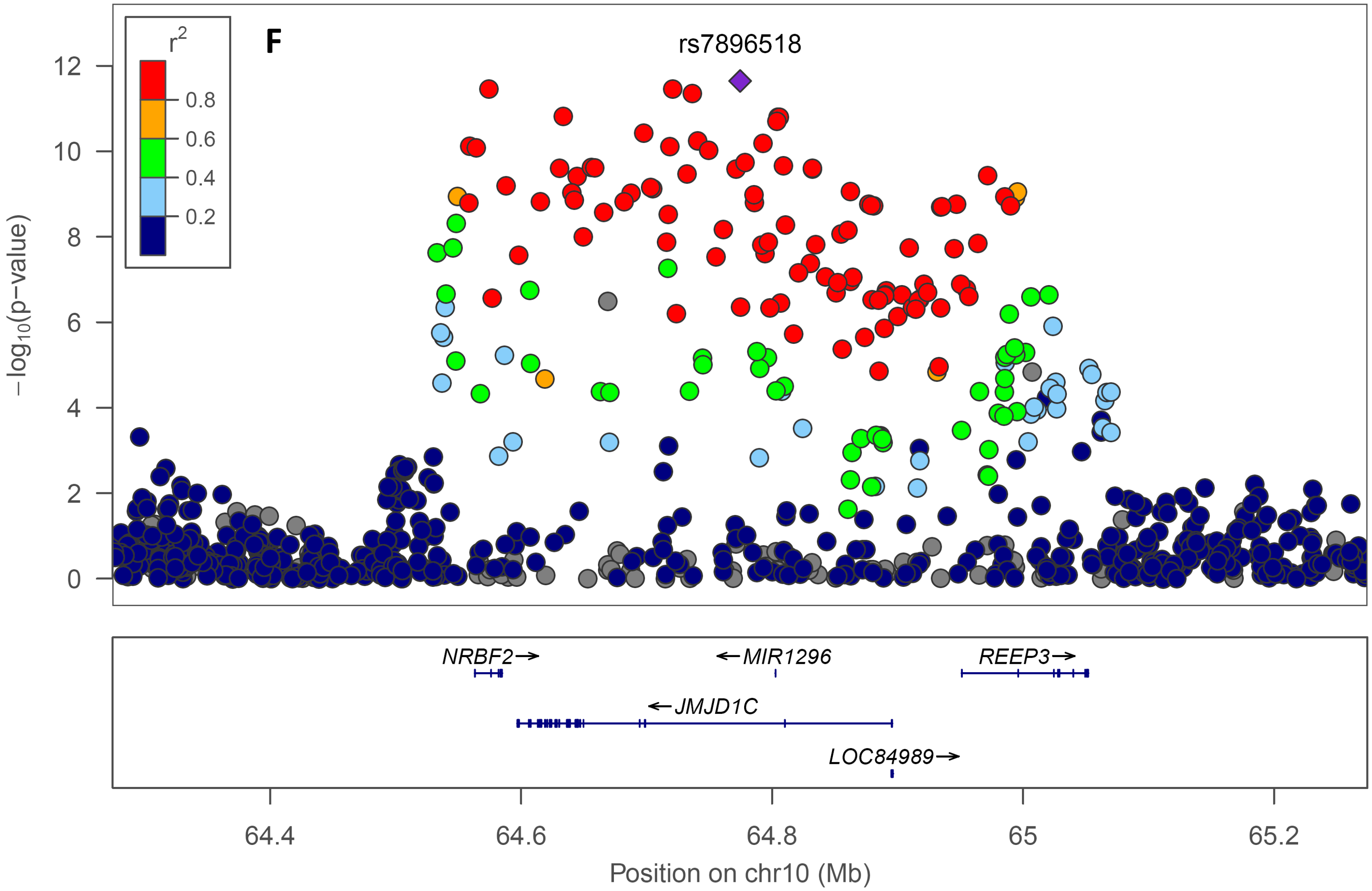

F
rs7896518
r2
0.8
0.6
0.4
0.2
12
10
8
6
4
2
0
−log10(p−value)
NRBF2→
←MIR1296
REEP3→
←JMJD1C
LOC84989→
64.4
64.6
64.8
65
65.2
Position on chr10 (Mb)

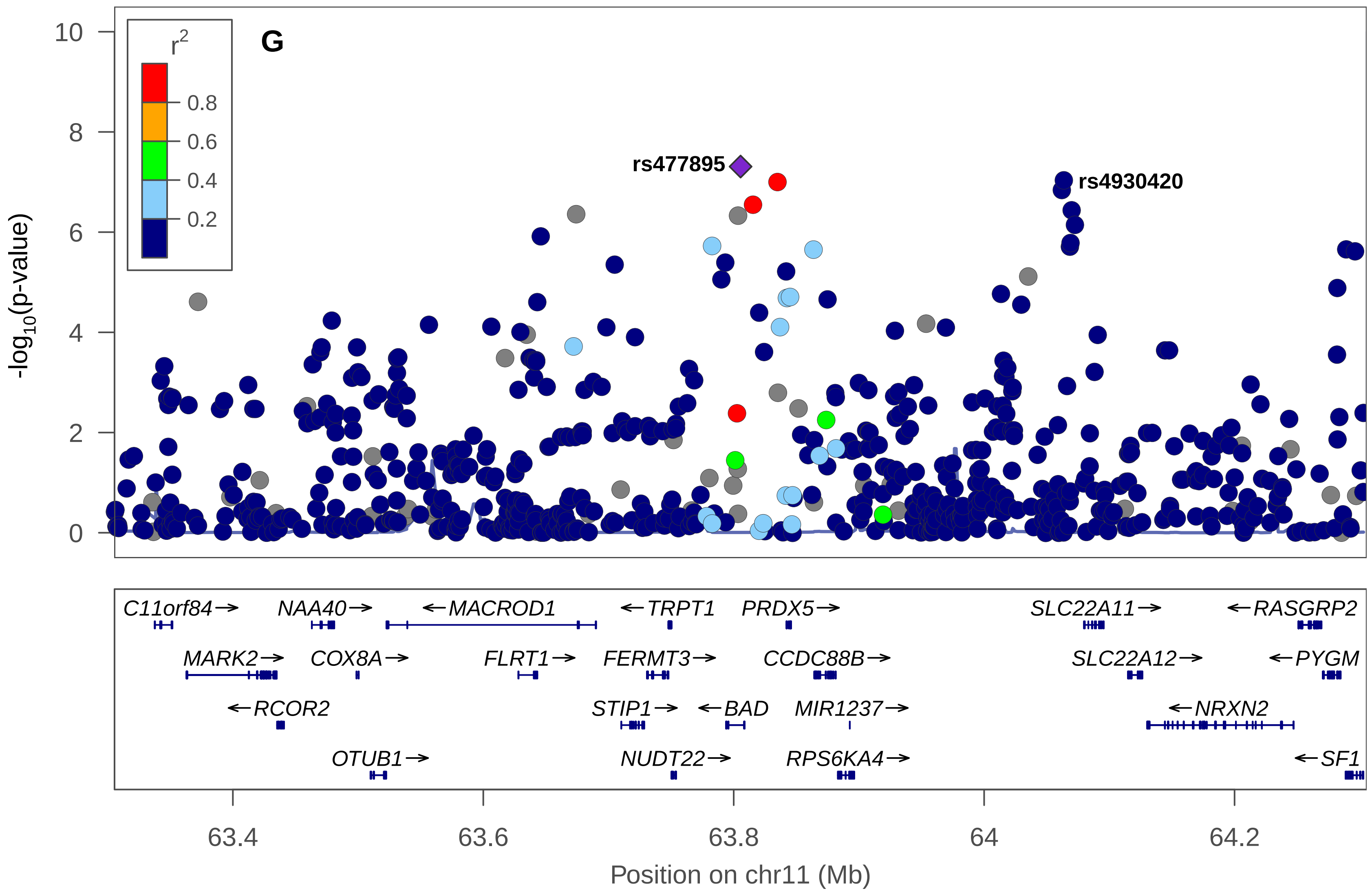
G
r2
0.8
0.6
0.4
0.2
rs477895
rs4930420
-log10(p-value)
10
8
6
4
2
0
C11orf84
NAA40
MACROD1
TRPT1
PRDX5
SLC22A11
RASGRP2
MARK2
COX8A
FLRT1
FERMT3
CCDC88B
SLC22A12
PYGM
RCOR2
STIP1
BAD
MIR1237
NRXN2
OTUB1
NUDT22
RPS6KA4
SF1
63.4
63.6
63.8
64
64.2
Position on chr11 (Mb)

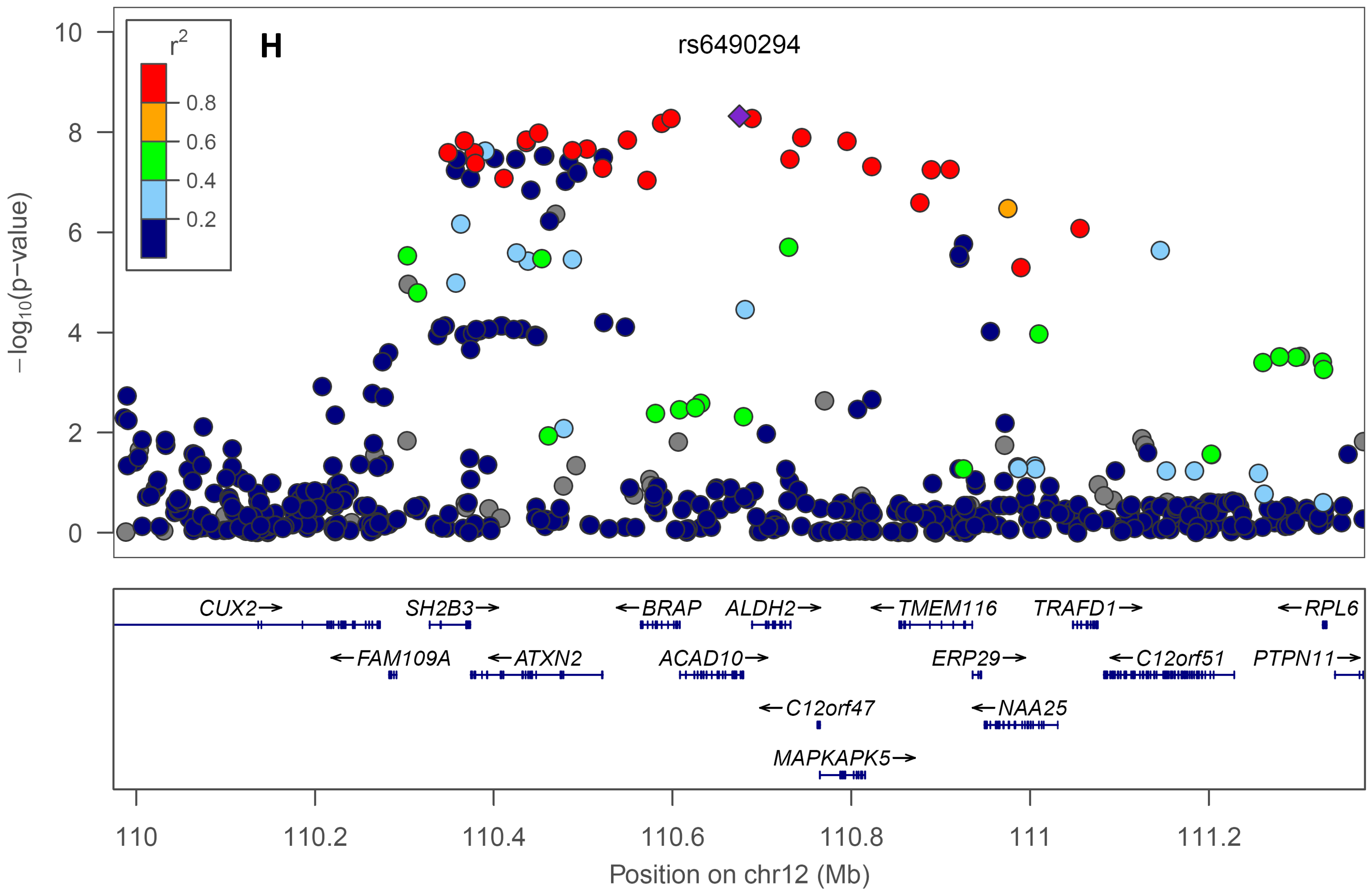

H
rs6490294
r2
0.8
0.6
0.4
0.2
−log10(p−value)
10
8
6
4
2
0
CUX2→
SH2B3→
←BRAP
ALDH2→
←TMEM116
TRAFD1→
←RPL6
←FAM109A
←ATXN2
ACAD10→
ERP29→
←C12orf51
PTPN11→
←C12orf47
←NAA25
MAPKAPK5→
110
110.2
110.4
110.6
110.8
111
111.2
Position on chr12 (Mb)

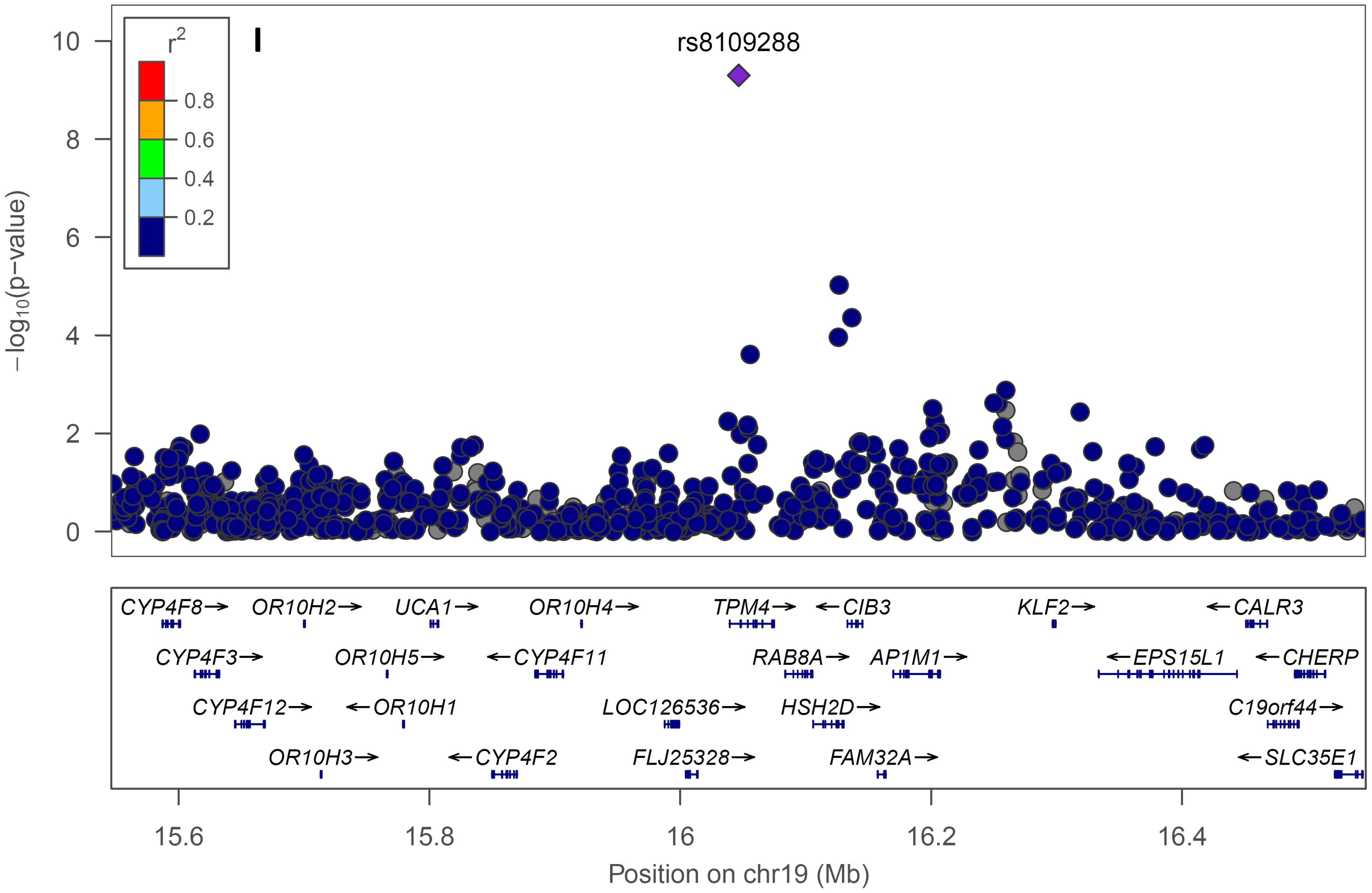

I
rs8109288
r2
0.8
0.6
0.4
0.2
−log10(p−value)
10
8
6
4
2
0
CYP4F8→
OR10H2→
UCA1→
OR10H4→
TPM4→
←CIB3
KLF2→
←CALR3
CYP4F3→
OR10H5→
←CYP4F11
RAB8A→
AP1M1→
←EPS15L1
←CHERP
CYP4F12→
←OR10H1
LOC126536→
HSH2D→
C19orf44→
OR10H3→
←CYP4F2
FLJ25328→
FAM32A→
←SLC35E1
15.6
15.8
16
16.2
16.4
Position on chr19 (Mb)

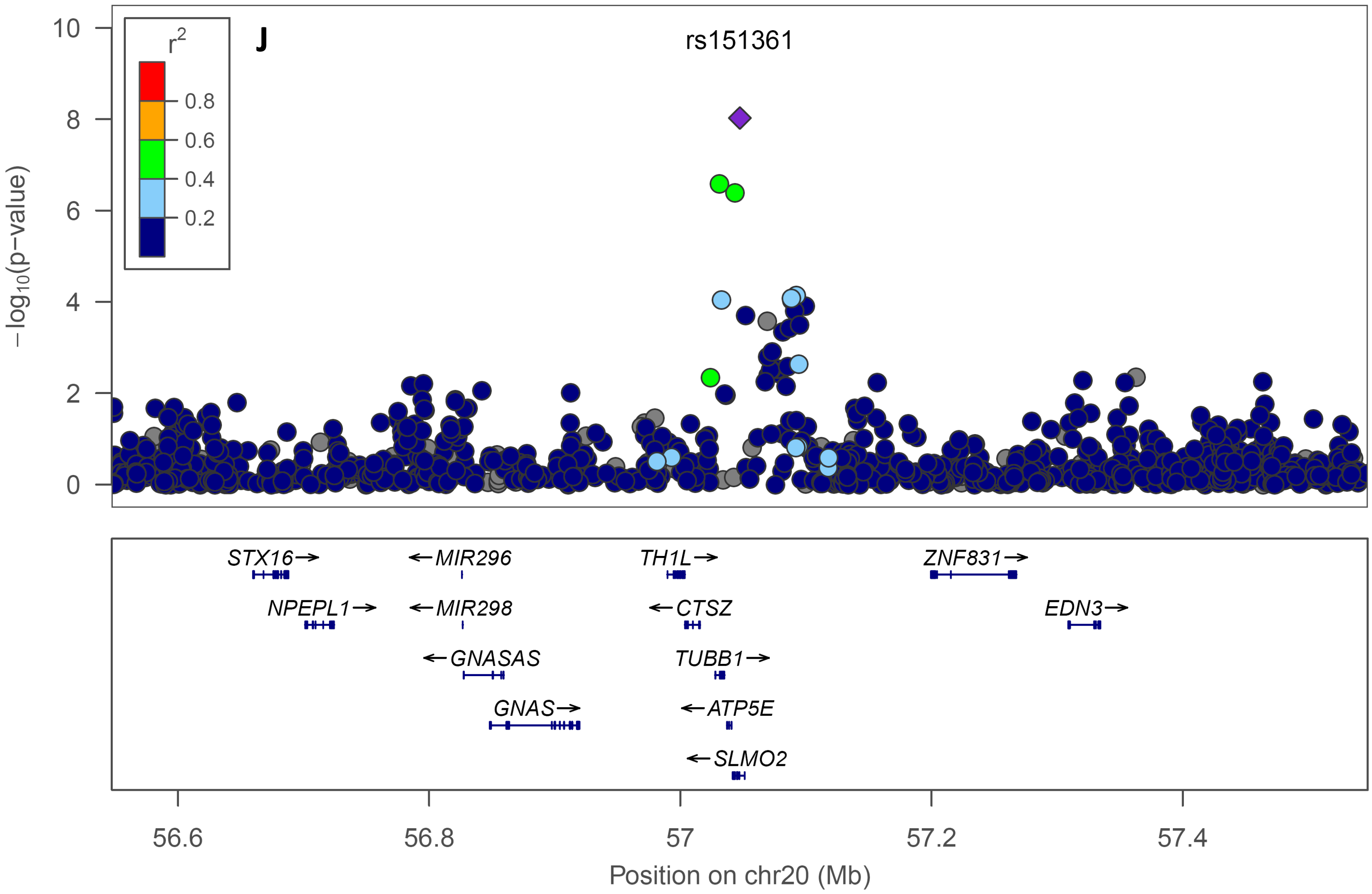

J
rs151361
r2
0.8
0.6
0.4
0.2
10
8
6
4
2
0
−log10(p−value)
STX16
NPEPL1
MIR296
MIR298
GNASAS
GNAS
TH1L
CTSZ
TUBB1
ATP5E
SLMO2
ZNF831
EDN3
56.6
56.8
57
57.2
57.4
Position on chr20 (Mb)
