# Supplementary material for: A Meta-Analysis and Genome-Wide Association Study of Platelet Count and Mean Platelet Volume in African Americans
Source: PLoS Genet. 2012 Mar 8;8(3):e1002491. doi: 10.1371/journal.pgen.1002491 (PMC3299192; doi:10.1371/journal.pgen.1002491)
Supplement: Figure S2 — Negative log(10) statistical significance plots of the each local region with 500 kbp on either side of the top SNP significantly associated with mean platelet volume. (PDF) [file pgen.1002491.s002.pdf]

Figure S2 (A-C): Negative log(10) statistical significance plots of the each local region with 500 kbp on either side of the top SNP significantly associated with mean platelet volume

Chromosomal position is on x-axis and –log(10) of p-value is on the y-axis. The color of a circle is based on the r^2 from HapMap II data. A gray circle represents a SNP for which r^2 data is not available from HapMap II. These plots were generated using LocusZoom (http://csg.sph.umich.edu/locuszoom/).

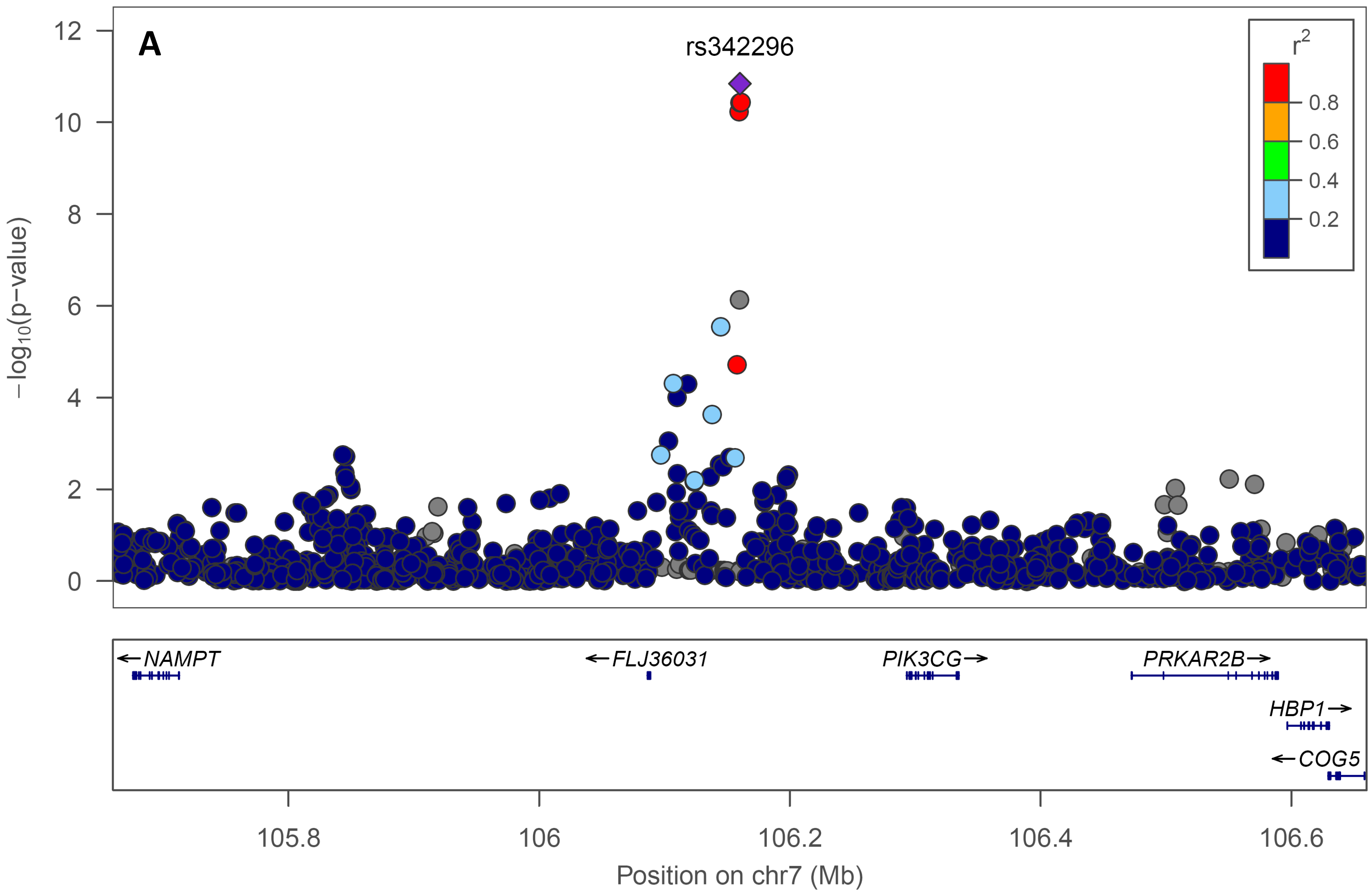
A
rs342296
r2
0.8
0.6
0.4
0.2
12
10
8
6
4
2
0
−log10(p−value)
NAMPT
FLJ36031
PIK3CG
PRKAR2B
HBP1
COG5
105.8
106
106.2
106.4
106.6
Position on chr7 (Mb)

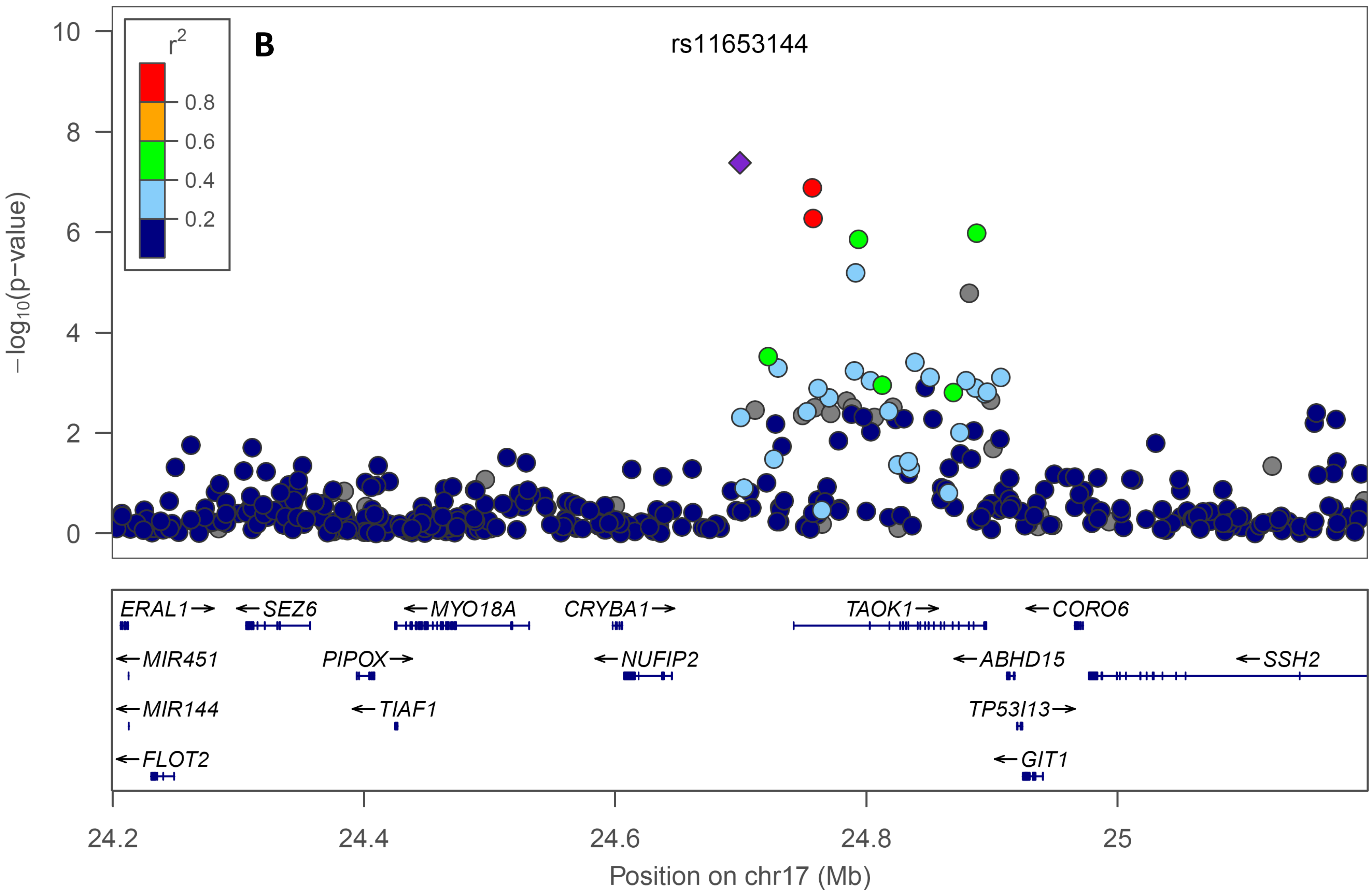
B
rs11653144
r2
0.8
0.6
0.4
0.2
−log10(p−value)
10
8
6
4
2
0
ERAL1→
←SEZ6
←MYO18A
CRYBA1→
TAOK1→
←CORO6
←MIR451
PIPOX→
←NUFIP2
←ABHD15
←SSH2
←MIR144
←TIAF1
TP53I13→
←FLOT2
←GIT1
24.2
24.4
24.6
24.8
25
Position on chr17 (Mb)

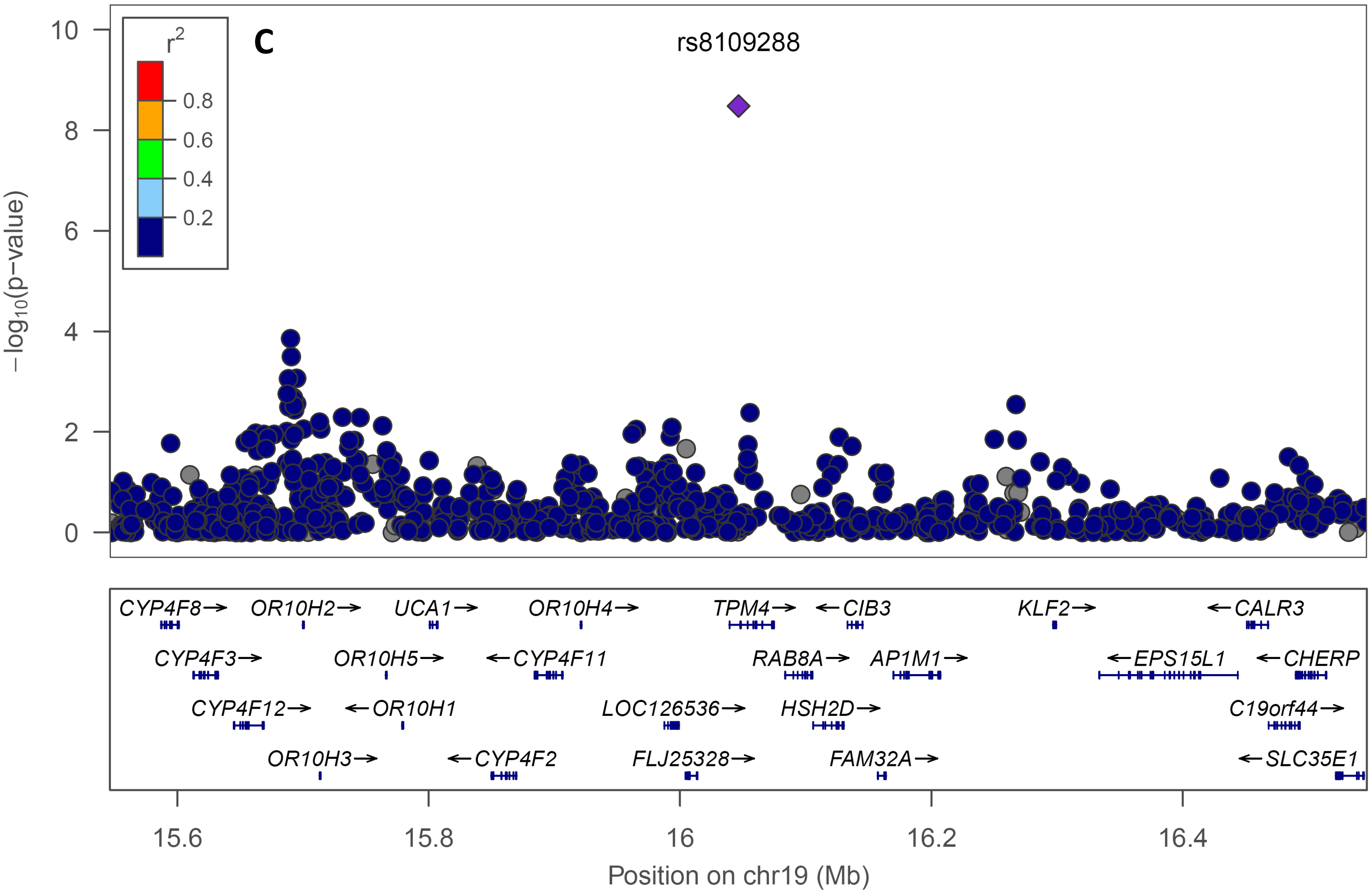

C
rs8109288
r2
0.8
0.6
0.4
0.2
−log10(p−value)
0
2
4
6
8
10
CYP4F8→
OR10H2→
UCA1→
OR10H4→
TPM4→
←CIB3
KLF2→
←CALR3
CYP4F3→
OR10H5→
←CYP4F11
RAB8A→
AP1M1→
←EPS15L1
←CHERP
CYP4F12→
←OR10H1
LOC126536→
HSH2D→
C19orf44→
OR10H3→
←CYP4F2
FLJ25328→
FAM32A→
←SLC35E1
15.6
15.8
16
16.2
16.4
Position on chr19 (Mb)
