# Supplementary material for: A Meta-Analysis and Genome-Wide Association Study of Platelet Count and Mean Platelet Volume in African Americans
Source: PLoS Genet. 2012 Mar 8;8(3):e1002491. doi: 10.1371/journal.pgen.1002491 (PMC3299192; doi:10.1371/journal.pgen.1002491)

Figure S4 (A-E): QQ plots of individual studies for mean platelet volume (MPV)

Blue dots are SNPs plotted on the x-axis of expected p-value under the null hypothesis against observed p-value in the study (p-values are plotted here as negative logarithm 10). Red diagonal line represents the line of unity, the region where expected and observed p-values are the same. Black lines above and below the red diagonal line bound 95% confidence intervals

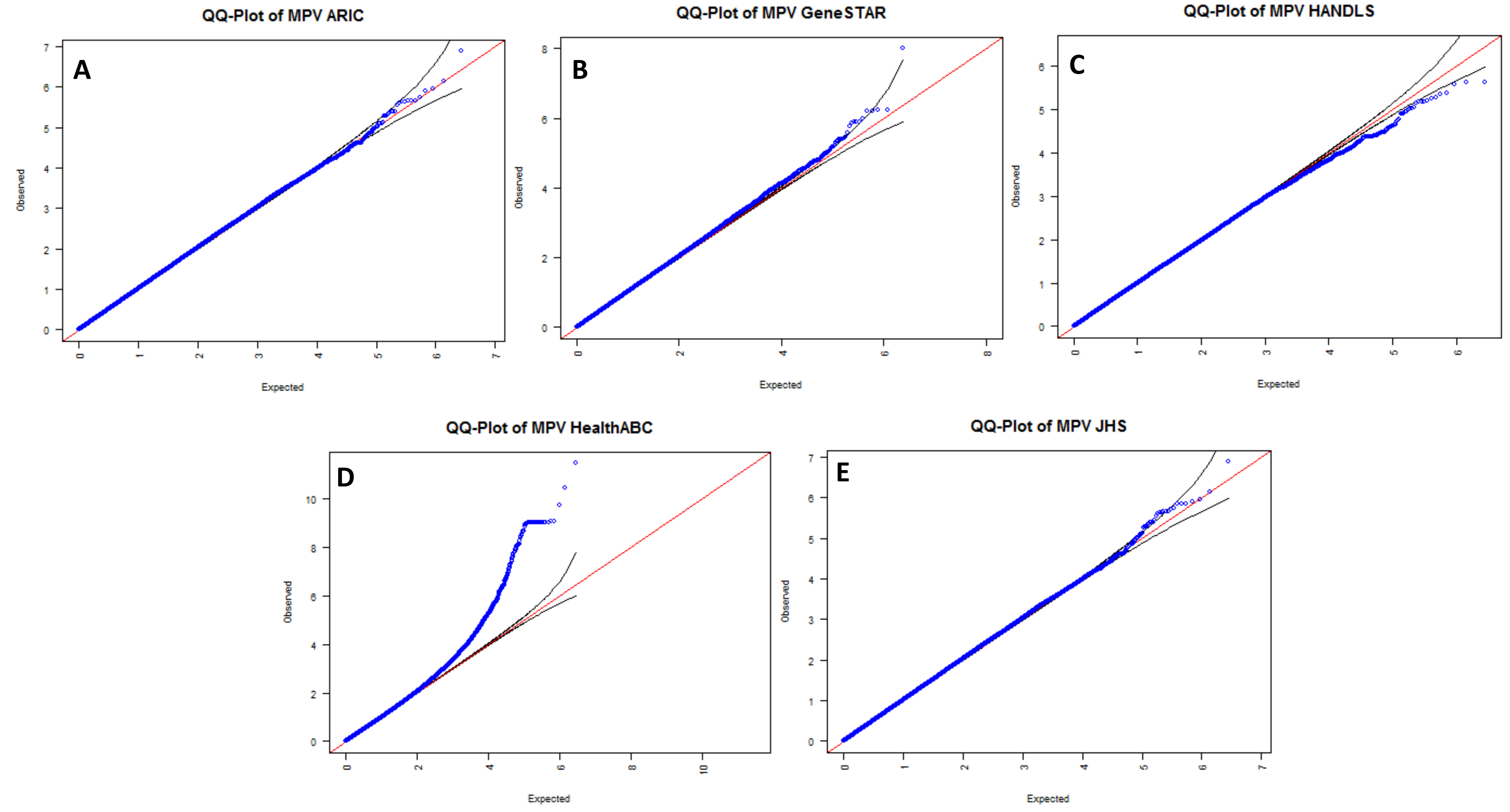

Supplement: Figure S4 — QQ plots of individual studies for mean platelet volume (MPV). (PDF) [file pgen.1002491.s004.pdf]
