# Supplementary material for: A Meta-Analysis and Genome-Wide Association Study of Platelet Count and Mean Platelet Volume in African Americans
Source: PLoS Genet. 2012 Mar 8;8(3):e1002491. doi: 10.1371/journal.pgen.1002491 (PMC3299192; doi:10.1371/journal.pgen.1002491)
Supplement: Table S1 — Genomic inflation factors for all GWAS analyses included in the meta-analysis. (PDF) [file pgen.1002491.s005.pdf]

Table S1: Genomic inflation factors for all GWAS analyses included in the meta-analysis

Study	Platelet count	MPV
**ARIC**	1.016	1.015
**CARDIA**	1.000	NA
**GeneSTAR**	1.031	1.03
**HANDLS**	0.994	0.98
**Health ABC**	1.012	1.03
**JHS**	1.125	1.01
**WHI**	1.060	NA

NA = not applicable
