# Supplementary material for: A Meta-Analysis and Genome-Wide Association Study of Platelet Count and Mean Platelet Volume in African Americans
Source: PLoS Genet. 2012 Mar 8;8(3):e1002491. doi: 10.1371/journal.pgen.1002491 (PMC3299192; doi:10.1371/journal.pgen.1002491)
Supplement: Table S2 — List of all SNPs with p-values<10−6 in the regions that were significant at GWAS threshold in platelet count meta-analysis. (PDF) [file pgen.1002491.s006.pdf]

Table S2: List of all SNPs with p-values < 10^{-6} in the regions that were significant at GWAS threshold in platelet count meta-analysis

SNP	Chr	Position	Gene	Role	Coded Allele	Allele Frequency	Effect Size (SE)	P-value	Het-P (I-square)	N
**rs214053**	6	25635942	LRRC16A	intron	T	56.6%	3.729 (0.712)	1.64×10^{-7}	0.37 (0)	16452
**rs214056**	6	25638063	LRRC16A	intron	C	43.4%	-3.719 (0.711)	1.72×10^{-7}	0.36 (0)	16452
**rs214057**	6	25639112	LRRC16A	intron	T	59.7%	3.578 (0.706)	4.04×10^{-7}	0.46 (0)	16452
**rs12526480**	6	25641513	LRRC16A	intron	T	69.5%	4.386 (0.763)	9.15×10^{-9}	0.62 (0)	16447
**rs214065**	6	25648906	LRRC16A	intron	A	40.2%	-3.511 (0.706)	6.71×10^{-7}	0.46 (0)	16389
**rs431659**	6	25653130	LRRC16A	intron	A	50.7%	3.134 (0.699)	7.28×10^{-6}	0.83 (0)	16452
**rs441460**	6	25656267	LRRC16A	intron	A	49.2%	-3.475 (0.689)	4.57×10^{-7}	0.68 (0)	16414
**rs368638**	6	25656552	LRRC16A	intron	A	50.9%	3.500 (0.690)	3.91×10^{-7}	0.69 (0)	16451
**rs118855**	6	25659861	LRRC16A	intron	A	47.2%	-3.248 (0.706)	4.18×10^{-6}	0.84 (0)	16452
**rs214048**	6	25661695	LRRC16A	intron	A	48.4%	-3.462 (0.695)	6.28×10^{-7}	0.82 (0)	16452
**rs214054**	6	25667877	LRRC16A	intron	A	52.9%	3.353 (0.697)	1.51×10^{-6}	0.76 (0)	16452
**rs12177652**	6	33586740			T	76.8%	-4.191 (0.842)	6.38×10^{-7}	0.08 (28.6)	16448
**rs210176**	6	33591894			A	60.8%	-3.759 (0.723)	2.02×10^{-7}	0.02 (46.5)	16452
**rs210179**	6	33593214			T	70.6%	4.621 (0.807)	1.03×10^{-8}	0.04 (39.2)	16445
**rs210180**	6	33593237			A	53.3%	3.666 (0.701)	1.73×10^{-7}	0.03 (42.3)	16452
**rs210188**	6	33599265			C	36.1%	-4.101 (0.748)	4.22×10^{-8}	0.04 (40.9)	16452
**rs449242**	6	33604692			T	36.9%	-4.408 (0.737)	2.26×10^{-9}	0.03 (43.6)	16452

**rs9469481**	6	33613572			A	55.1%	3.586 (0.701)	3.15×10^{-7}	0.03 (44.6)	16452
**rs380163**	6	33613666			A	44.5%	-3.583 (0.701)	3.23×10^{-7}	0.02 (44.7)	16452
**rs367408**	6	33613724			A	55.5%	3.58 (0.701)	3.25×10^{-7}	0.02 (45)	16452
**rs368716**	6	33613959			T	44.9%	-3.584 (0.700)	3.08×10^{-7}	0.02 (45.6)	16452
**rs396516**	6	33614045			A	54.9%	3.622 (0.705)	2.77×10^{-7}	0.02 (47)	16256
**rs367897**	6	33614220			C	55.5%	3.628 (0.702)	2.36×10^{-7}	0.06 (33.5)	16369
**rs404778**	6	33614543			T	44.5%	-3.632 (0.701)	2.17×10^{-7}	0.02 (45.4)	16452
**rs9469483**	6	33615234			C	55.5%	3.658 (0.701)	1.81×10^{-7}	0.02 (45.4)	16452
**rs210197**	6	33615969			T	52.5%	-3.543 (0.701)	4.3×10^{-7}	0.76 (0)	16415
**rs9469485**	6	33622071			T	30.6%	4.713 (0.787)	2.09×10^{-9}	0.18 (9.2)	16452
**rs210152**	6	33623498			A	88.5%	6.551 (1.183)	3.07×10^{-8}	0.72 (0)	16450
**rs9366824**	6	33629555			T	30.1%	5.098 (0.761)	2.05×10^{-11}	0.62 (0)	16452
**rs210170**	6	33633658			T	30.1%	-4.093 (0.756)	6.2×10^{-8}	0.54 (0)	16451
**rs210130**	6	33641382			T	41.7%	4.632 (0.708)	5.91×10^{-11}	0.65 (0)	16452
**rs6910233**	6	33642704			T	57.0%	3.769 (0.704)	8.57×10^{-8}	0.32 (0)	16410
**rs6914422**	6	33642858			T	29.8%	5.116 (0.761)	1.74×10^{-11}	0.62 (0)	16408
**rs210132**	6	33644648			T	42.6%	4.786 (0.700)	8.11×10^{-12}	0.24 (0)	16414
**rs210133**	6	33644775			C	35.7%	-4.733 (0.721)	5.33×10^{-11}	0.39 (0)	16278
**rs210134**	6	33648187	BAK1	near-gene-3	A	28.6%	-6.156 (0.777)	2.32×10^{-15}	0.18 (10.6)	16452
**rs210135**	6	33648670	BAK1	intron	A	71.7%	5.988 (0.784)	2.18×10^{-14}	0.31 (0)	16388

**rs513349**	6	33649697	BAK1	intron	A	37.3%	-5.226 (0.725)	5.69×10^{-13}	0.23 (2)	16451
**rs9296095**	6	33650501	BAK1	intron	T	71.3%	-5.429 (0.772)	2.03×10^{-12}	0.41 (0)	16417
**rs210139**	6	33651387	BAK1	intron	A	53.0%	-4.985 (0.691)	5.44×10^{-13}	0.17 (10.9)	16411
**rs5745582**	6	33654476	BAK1	intron	T	28.7%	5.397 (0.770)	2.48×10^{-12}	0.47 (0)	16450
**rs210142**	6	33654815	BAK1	intron	T	26.0%	-5.010 (0.818)	8.32×10^{-10}	0.002 (61.2)	16452
**rs530878**	6	33659492	GGNBP1	near-gene-5	A	47.7%	-4.569 (0.724)	2.72×10^{-10}	0.24 (0.2)	16452
**rs6900408**	6	33660142	GGNBP1	near-gene-3	T	42.8%	4.562 (0.716)	1.86×10^{-10}	0.19 (7.7)	16452
**rs549888**	6	33660180	FLJ43752	intron	A	36.2%	-5.549 (0.736)	4.7×10^{-14}	0.12 (21.5)	16452
**rs7772031**	6	135301427	ALDH8A1	intron	A	55.2%	3.197 (0.714)	7.49×10^{-6}	0.21 (4.5)	16452
**rs13220662**	6	135437241			A	58.4%	-4.156 (0.762)	5.01×10^{-8}	0.17 (11.7)	16444
**rs7775698**	6	135460328			T	21.1%	4.008 (0.901)	8.68×10^{-6}	0.55 (0)	16452
**rs4895441**	6	135468266			A	90.2%	-5.795 (1.166)	6.72×10^{-7}	1 (0)	16415
**rs9376092**	6	135468837			A	19.6%	4.242 (0.868)	1.03×10^{-6}	0.55 (0)	16410
**rs9389269**	6	135468852			T	91.9%	-7.040 (1.267)	2.66×10^{-8}	0.98 (0)	16452
**rs9402686**	6	135469510			A	8.1%	7.236 (1.312)	3.51×10^{-8}	0.99 (0)	16452
**rs9494145**	6	135474245			T	92.7%	-8.187 (1.377)	2.79×10^{-9}	0.99 (0)	16449
**rs9483788**	6	135477194			T	92.3%	-7.584 (1.298)	5.21×10^{-9}	1 (0)	16452
**rs6961069**	7	80056897			T	51.1%	3.439 (0.690)	8.89×10^{-7}	0.61 (0)	16452
**rs1194182**	7	80069440	CD36	near-gene-5	C	67.1%	3.926 (0.752)	1.79×10^{-7}	0.61 (0)	16452
**rs17154155**	7	80072179	CD36	intron	T	45.4%	4.031 (0.705)	1.1×10^{-8}	0.81 (0)	16452

**rs13236689**	7	80073950	CD36	intron	T	56.4%	-4.181 (0.704)	2.84 x 10^{-9}	0.73 (0)	16443
**rs1761661**	7	80075977	CD36	intron	A	33.5%	-3.606 (0.738)	1.02 x 10^{-6}	0.68 (0)	16452
**rs1722505**	7	80078625	CD36	intron	T	33.5%	-3.319 (0.730)	7.24 x 10^{-6}	0.54 (0)	16452
**rs1722502**	7	80079810	CD36	intron	A	33.5%	-3.471 (0.746)	3.24 x 10^{-6}	0.55 (0)	16452
**rs4731643**	7	80085965	CD36	intron	A	36.7%	-3.457 (0.755)	4.74 x 10^{-6}	0.23 (0)	14615
**rs3211913**	7	80132540	CD36	intron	A	66.9%	3.890 (0.862)	6.47 x 10^{-6}	0.01 (56.8)	16452
**rs17154246**	7	80133170	CD36	intron	A	24.3%	-4.297 (0.909)	2.27 x 10^{-6}	0.13 (19.2)	16452
**rs342292**	7	106157880			C	66.6%	3.604 (0.755)	1.83 x 10^{-6}	0.27 (0)	16452
**rs342293**	7	106159455			C	61.4%	4.055 (0.717)	1.58 x 10^{-8}	0.19 (9)	16411
**rs342294**	7	106159858			T	61.4%	3.841 (0.861)	8.19 x 10^{-6}	0.35 (0)	11068
**rs342295**	7	106159996			T	37.7%	-4.077 (0.746)	4.21 x 10^{-8}	0.21 (5.3)	16452
**rs342296**	7	106160139			A	37.6%	-4.104 (0.738)	2.68 x 10^{-8}	0.2 (6.1)	16451
**rs342298**	7	106160882			T	37.3%	-4.156 (0.752)	3.31 x 10^{-8}	0.27 (0)	16452
**rs10761703**	10	64532968			A	45.1%	3.960 (0.711)	2.34 x 10^{-8}	0.77 (0)	16452
**rs10822133**	10	64535878			A	30.6%	3.642 (0.762)	1.73 x 10^{-6}	0.003 (59.6)	16452
**rs10761708**	10	64538046			A	55.3%	3.391 (0.717)	2.23 x 10^{-6}	0.63 (0)	16452
**rs10995441**	10	64539245			T	54.8%	3.568 (0.707)	4.44 x 10^{-7}	0.67 (0)	16452
**rs10761711**	10	64539948			A	50.5%	-3.633 (0.700)	2.13 x 10^{-7}	0.67 (0)	16415
**rs4414112**	10	64545552			A	58.6%	-4.172 (0.741)	1.77 x 10^{-8}	0.16 (14.2)	16445
**rs7901794**	10	64547944			T	54.5%	3.380 (0.759)	7.98 x 10^{-6}	0.19 (8.4)	16452

**rs1396964**	10	64548304			A	39.7%	4.271 (0.729)	4.74×10^{-9}	0.13 (19.2)	16452
**rs1553789**	10	64549235			T	36.6%	4.431 (0.728)	1.13×10^{-9}	0.31 (0)	16395
**rs7082200**	10	64558327			C	59.3%	-4.375 (0.725)	1.58×10^{-9}	0.29 (0)	16452
**rs10995450**	10	64558917			T	59.5%	-4.653 (0.715)	7.47×10^{-11}	0.32 (0)	16414
**rs871690**	10	64564065	NRBF2	intron	A	59.5%	-4.642 (0.714)	8.12×10^{-11}	0.32 (0)	16451
**rs7073746**	10	64574077	NRBF2	intron	A	66.3%	-5.091 (0.731)	3.41×10^{-12}	0.05 (35.1)	16452
**rs10733787**	10	64576844	NRBF2	intron	A	41.9%	3.662 (0.712)	2.71×10^{-7}	0.001 (64.5)	16452
**rs1009984**	10	64586475			T	44.9%	-3.181 (0.702)	5.87×10^{-6}	0.56 (0)	16414
**rs10733788**	10	64587871			T	58.3%	-4.397 (0.711)	6.31×10^{-10}	0.26 (0)	16452
**rs1935**	10	64597829	JMJD1C	missense	C	64.5%	-4.071 (0.732)	2.66×10^{-8}	0.0002 (70.1)	16452
**rs907**	10	64606685	JMJD1C	intron	A	51.3%	-3.654 (0.699)	1.76×10^{-7}	0.08 (30.2)	16405
**rs2306263**	10	64607421	JMJD1C	intron	T	38.7%	-3.212 (0.724)	9.08×10^{-6}	0.03 (41.3)	16452
**rs3211105**	10	64615370	JMJD1C	reference	A	41.6%	4.304 (0.712)	1.48×10^{-9}	0.27 (0)	16451
**rs10465990**	10	64630542	JMJD1C	intron	A	39.6%	4.534 (0.716)	2.48×10^{-10}	0.43 (0)	16452
**rs4379723**	10	64633455	JMJD1C	intron	T	66.5%	-4.936 (0.731)	1.5×10^{-11}	0.1 (25.7)	16452
**rs1579045**	10	64640454	JMJD1C	intron	A	41.6%	4.359 (0.712)	9.17×10^{-10}	0.23 (1.8)	16448
**rs7920159**	10	64642182	JMJD1C	intron	C	58.3%	-4.315 (0.712)	1.37×10^{-9}	0.29 (0)	16452
**rs10761725**	10	64644543	JMJD1C	missense	A	60.4%	-4.487 (0.717)	3.81×10^{-10}	0.52 (0)	16425
**rs10822148**	10	64649253	JMJD1C	intron	A	58.3%	-4.158 (0.726)	9.96×10^{-9}	0.43 (0)	16452
**rs4335432**	10	64655823	JMJD1C	intron	T	39.6%	4.540 (0.717)	2.36×10^{-10}	0.46 (0)	16452

**rs6479889**	10	64658633	JMJD1C	intron	A	39.6%	4.538 (0.716)	2.41×10^{-10}	0.46 (0)	16440
**rs10761728**	10	64665574	JMJD1C	intron	T	41.7%	4.240 (0.712)	2.62×10^{-9}	0.41 (0)	16452
**rs7082066**	10	64668977	JMJD1C	intron	A	51.0%	-3.892 (0.761)	3.19×10^{-7}	0.06 (31.5)	13729
**rs7081614**	10	64681854	JMJD1C	intron	T	41.4%	4.304 (0.712)	1.48×10^{-9}	0.33 (0)	16446
**rs10822150**	10	64687502	JMJD1C	intron	A	42.0%	4.374 (0.715)	9.38×10^{-10}	0.33 (0)	16452
**rs10761731**	10	64697616	JMJD1C	intron	A	67.6%	-4.957 (0.749)	3.66×10^{-11}	0.25 (0)	16452
**rs7909555**	10	64703114	JMJD1C	intron	T	58.4%	-4.392 (0.712)	6.96×10^{-10}	0.28 (0)	16452
**rs10761733**	10	64704790	JMJD1C	intron	T	58.2%	-4.388 (0.712)	7.3×10^{-10}	0.29 (0)	16452
**rs4417159**	10	64715704	JMJD1C	intron	A	41.8%	4.109 (0.723)	1.31×10^{-8}	0.48 (0)	16452
**rs7477138**	10	64716674	JMJD1C	intron	A	43.4%	-3.881 (0.714)	5.39×10^{-8}	0.03 (43.1)	16452
**rs10995493**	10	64717032	JMJD1C	intron	T	41.8%	4.231 (0.713)	2.96×10^{-9}	0.47 (0)	16452
**rs7080386**	10	64718312	JMJD1C	intron	A	32.5%	4.896 (0.752)	7.62×10^{-11}	0.27 (0)	16452
**rs7075195**	10	64720665	JMJD1C	intron	A	67.6%	-5.134 (0.738)	3.4×10^{-12}	0.15 (14.5)	16452
**rs7913332**	10	64723656	JMJD1C	intron	A	58.2%	-3.555 (0.713)	6.26×10^{-7}	0.002 (61.3)	16452
**rs10761739**	10	64732014	JMJD1C	intron	C	32.5%	4.664 (0.742)	3.37×10^{-10}	0.13 (19.3)	16403
**rs10761741**	10	64736192	JMJD1C	intron	T	32.3%	5.106 (0.737)	4.34×10^{-12}	0.16 (12.8)	16407
**rs7070296**	10	64740444	JMJD1C	intron	A	35.0%	4.820 (0.735)	5.56×10^{-11}	0.09 (26.4)	16452
**rs10400028**	10	64744349	JMJD1C	intron	A	61.4%	3.254 (0.723)	6.78×10^{-6}	0.03 (43.9)	16452
**rs10995499**	10	64744907	JMJD1C	intron	A	38.3%	-3.221 (0.728)	9.64×10^{-6}	0.03 (42.5)	16343
**rs7088045**	10	64749403	JMJD1C	intron	A	38.7%	4.653 (0.718)	9.36×10^{-11}	0.61 (0)	16452

**rs10761742**	10	64755054	JMJD1C	intron	A	66.4%	-4.053 (0.731)	2.9×10^{-8}	0.001 (65)	16452
**rs10995505**	10	64761165	JMJD1C	intron	A	60.3%	-4.223 (0.728)	6.68×10^{-9}	0.43 (0)	16414
**rs10740118**	10	64771213	JMJD1C	intron	C	32.3%	4.746 (0.751)	2.57×10^{-10}	0.12 (20.8)	16415
**rs7896518**	10	64774506	JMJD1C	intron	A	67.6%	-5.178 (0.738)	2.26×10^{-12}	0.14 (16.4)	16452
**rs10761746**	10	64774900	JMJD1C	intron	A	58.3%	-3.605 (0.714)	4.39×10^{-7}	0.002 (61.3)	16452
**rs10761747**	10	64778162	JMJD1C	intron	C	39.7%	4.572 (0.717)	1.82×10^{-10}	0.52 (0)	16409
**rs7098614**	10	64785433	JMJD1C	intron	T	39.0%	4.442 (0.727)	1.02×10^{-9}	0.48 (0)	16452
**rs7083356**	10	64785794	JMJD1C	intron	A	41.1%	4.319 (0.715)	1.55×10^{-9}	0.36 (0)	16452
**rs7900050**	10	64787776	JMJD1C	intron	A	38.4%	-3.307 (0.723)	4.82×10^{-6}	0.02 (45)	16452
**rs12355784**	10	64791571	JMJD1C	intron	A	33.7%	4.141 (0.732)	1.54×10^{-8}	0.004 (58.8)	16392
**rs9629895**	10	64792793	JMJD1C	intron	A	59.1%	-4.674 (0.716)	6.48×10^{-11}	0.4 (0)	16383
**rs10822163**	10	64794104	JMJD1C	intron	C	66.3%	-4.110 (0.738)	2.41×10^{-8}	0.001 (63.1)	16452
**rs12256924**	10	64796074	JMJD1C	intron	A	38.7%	-3.258 (0.723)	6.67×10^{-6}	0.03 (41.6)	16451
**rs6479896**	10	64796838	JMJD1C	intron	T	66.4%	-4.156 (0.731)	1.32×10^{-8}	0.003 (59.7)	16452
**rs7909692**	10	64798283	JMJD1C	intron	A	41.6%	3.599 (0.714)	4.57×10^{-7}	0.003 (59.7)	16452
**rs7923609**	10	64803828	JMJD1C	intron	A	66.5%	-4.911 (0.732)	1.94×10^{-11}	0.13 (18.4)	16451
**rs2893919**	10	64804784	JMJD1C	intron	A	33.5%	4.944 (0.733)	1.58×10^{-11}	0.13 (19.4)	16452
**rs2393966**	10	64804820	JMJD1C	intron	T	66.5%	-4.944 (0.733)	1.58×10^{-11}	0.13 (19.4)	16452
**rs7076310**	10	64805678	JMJD1C	intron	A	33.5%	4.946 (0.734)	1.58×10^{-11}	0.13 (19.4)	16452
**rs6479897**	10	64806634	JMJD1C	intron	T	41.5%	3.644 (0.716)	3.53×10^{-7}	0.004 (58.8)	16452

**rs7910927**	10	64808916	JMJD1C	intron	T	66.5%	-4.736 (0.746)	2.16×10^{-10}	0.21 (5.2)	16452
**rs2393969**	10	64810446	JMJD1C	intron	A	66.6%	-4.284 (0.734)	5.22×10^{-9}	0.01 (56)	16412
**rs10822165**	10	64816965	JMJD1C	intron	T	59.6%	-3.477 (0.729)	1.85×10^{-6}	0.01 (56.6)	16452
**rs7095571**	10	64820965	JMJD1C	intron	T	67.7%	-4.043 (0.749)	6.79×10^{-8}	0.003 (60.3)	16452
**rs10761752**	10	64830327	JMJD1C	intron	T	67.8%	-4.116 (0.750)	4.1×10^{-8}	0.01 (55.9)	16452
**rs7912893**	10	64832006	JMJD1C	intron	A	32.1%	4.759 (0.752)	2.47×10^{-10}	0.09 (27.4)	16452
**rs7896783**	10	64832159	JMJD1C	intron	A	32.1%	4.753 (0.752)	2.58×10^{-10}	0.09 (27.4)	16452
**rs10740123**	10	64834521	JMJD1C	intron	A	60.0%	-4.123 (0.728)	1.51×10^{-8}	0.36 (0)	16452
**rs10761756**	10	64842334	JMJD1C	intron	T	32.1%	4.010 (0.751)	8.59×10^{-8}	0.002 (60.8)	16452
**rs6479901**	10	64850782	JMJD1C	intron	A	40.0%	3.855 (0.742)	2.03×10^{-7}	0.31 (0)	16452
**rs7923544**	10	64852262	JMJD1C	intron	T	68.1%	-3.983 (0.752)	1.16×10^{-7}	0.002 (61.1)	16452
**rs10761762**	10	64854723	JMJD1C	intron	T	68.1%	-4.377 (0.760)	8.49×10^{-9}	0.08 (30)	16452
**rs10740124**	10	64855800	JMJD1C	intron	T	39.7%	3.369 (0.732)	4.22×10^{-6}	0.01 (55.2)	16452
**rs10761766**	10	64860333	JMJD1C	intron	A	31.6%	4.441 (0.767)	6.95×10^{-9}	0.1 (24.7)	16452
**rs3740331**	10	64862294	JMJD1C	intron	A	31.5%	4.627 (0.754)	8.53×10^{-10}	0.07 (32)	16452
**rs10761767**	10	64862437	JMJD1C	intron	T	60.5%	-3.873 (0.729)	1.09×10^{-7}	0.38 (0)	16452
**rs10761768**	10	64864135	JMJD1C	intron	A	39.3%	3.899 (0.729)	8.75×10^{-8}	0.33 (0)	16452
**rs3999089**	10	64873814	JMJD1C	intron	A	70.4%	-3.673 (0.776)	2.23×10^{-6}	0.06 (35)	16412
**rs10509186**	10	64877024	JMJD1C	intron	T	31.1%	4.533 (0.752)	1.7×10^{-9}	0.06 (33.5)	16452
**rs7085621**	10	64878932	JMJD1C	intron	T	68.8%	-4.526 (0.753)	1.82×10^{-9}	0.06 (33.3)	16452

**rs10740125**	10	64879615	JMJD1C	intron	T	68.8%	-3.852 (0.751)	2.9 x 10^{-7}	0.005 (57.2)	16452
**rs10740126**	10	64880941	JMJD1C	intron	A	68.8%	-4.524 (0.753)	1.86 x 10^{-9}	0.06 (33.1)	16452
**rs7092784**	10	64884755	JMJD1C	intron	T	31.3%	3.846 (0.751)	3.03 x 10^{-7}	0.005 (57.1)	16452
**rs7074735**	10	64889285	JMJD1C	intron	A	39.5%	3.574 (0.740)	1.38 x 10^{-6}	0.29 (0)	16452
**rs7075205**	10	64889600	JMJD1C	intron	T	61.0%	-3.797 (0.735)	2.36 x 10^{-7}	0.29 (0)	16452
**rs10995540**	10	64889936	JMJD1C	intron	C	60.5%	-3.772 (0.728)	2.24 x 10^{-7}	0.32 (0)	16452
**rs10159609**	10	64890934	JMJD1C	intron	T	39.5%	3.799 (0.728)	1.79 x 10^{-7}	0.3 (0)	16452
**rs10761771**	10	64900170	LOC100128604	intron	T	68.0%	-3.747 (0.756)	7.14 x 10^{-7}	0.002 (61.4)	16452
**rs10995541**	10	64903341	LOC100128604	intron	T	39.4%	3.769 (0.728)	2.24 x 10^{-7}	0.33 (0)	16415
**rs7909960**	10	64909183	LOC100128604	intron	A	31.3%	4.302 (0.764)	1.78 x 10^{-8}	0.1 (25.6)	16452
**rs2393978**	10	64911816	LOC100128604	intron	A	39.4%	3.727 (0.738)	4.49 x 10^{-7}	0.32 (0)	16452
**rs7915779**	10	64914250	LOC100128604	intron	C	31.2%	3.779 (0.751)	4.88 x 10^{-7}	0.002 (61.3)	16452
**rs7085862**	10	64916453	LOC100128604	intron	T	60.6%	-3.720 (0.727)	3.07 x 10^{-7}	0.35 (0)	16452
**rs2393977**	10	64917615	LOC100128604	intron	A	68.8%	-3.855 (0.751)	2.84 x 10^{-7}	0.01 (55.3)	16452
**rs10740129**	10	64920814	LOC100128604	intron	A	29.5%	4.161 (0.788)	1.28 x 10^{-7}	0.05 (37.5)	16452
**rs7910662**	10	64923706	LOC100128604	intron	T	39.3%	3.787 (0.728)	1.96 x 10^{-7}	0.31 (0)	16376
**rs10509189**	10	64934132	LOC100128604	intron	T	68.8%	-3.788 (0.751)	4.57 x 10^{-7}	0.002 (61.3)	16452
**rs4486511**	10	64934272	LOC100128604	intron	T	31.3%	4.514 (0.752)	1.98 x 10-9	0.06 (33.2)	16452
**rs9971352**	10	64935114	LOC100128604	intron	A	68.8%	-4.516 (0.752)	1.94 x 10^{-9}	0.06 (33.3)	16452
**rs10761779**	10	64944933	LOC100128604	intron	A	68.5%	-4.316 (0.768)	1.89 x 10^{-8}	0.08 (28.8)	16249

**rs7082470**	10	64947032	LOC100128604	intron	A	31.0%	4.553 (0.756)	1.7×10^{-9}	0.07 (31.4)	16452
**rs10822179**	10	64950170	REEP3	near-gene-5	T	61.1%	-3.869 (0.732)	1.26×10^{-7}	0.35 (0)	16452
**rs10761781**	10	64954609	REEP3	intron	T	59.1%	-3.682 (0.703)	1.64×10^{-7}	0.25 (0)	16401
**rs7085018**	10	64956673	REEP3	intron	T	68.7%	-3.877 (0.751)	2.43×10^{-7}	0.002 (61)	16452
**rs7920036**	10	64963866	REEP3	intron	T	68.6%	-4.327 (0.763)	1.4×10^{-8}	0.08 (28.8)	16452
**rs7897379**	10	64971731	REEP3	intron	T	66.5%	-4.667 (0.744)	3.61×10^{-10}	0.1 (23.9)	16452
**rs6479905**	10	64985237	REEP3	intron	A	48.6%	3.163 (0.701)	6.51×10^{-6}	0.01 (53.7)	16452
**rs10740134**	10	64985439	REEP3	intron	T	67.1%	-4.579 (0.752)	1.15×10^{-9}	0.14 (16.6)	16452
**rs7919685**	10	64985806	REEP3	intron	T	51.1%	-3.155 (0.709)	8.69×10^{-6}	0.01 (55.8)	16452
**rs12247907**	10	64987051	REEP3	intron	C	51.5%	-3.180 (0.701)	5.76×10^{-6}	0.01 (53.6)	16452
**rs7070761**	10	64987062	REEP3	intron	A	48.5%	3.184 (0.701)	5.57×10^{-6}	0.01 (53.6)	16452
**rs10761785**	10	64988772	REEP3	intron	T	42.4%	3.584 (0.710)	6.39×10^{-7}	0.02 (46.5)	16452
**rs2393986**	10	64990012	REEP3	intron	A	67.0%	-4.526 (0.753)	1.87×10^{-9}	0.17 (12.3)	16452
**rs7899657**	10	64993271	REEP3	intron	A	50.9%	-3.255 (0.705)	3.9×10^{-6}	0.01 (53.7)	16452
**rs10733793**	10	64993815	REEP3	intron	T	66.3%	-4.573 (0.752)	1.2×10^{-9}	0.19 (9.1)	16452
**rs4746203**	10	64994003	REEP3	intron	T	50.8%	-3.197 (0.704)	5.68×10^{-6}	0.01 (52.3)	16451
**rs10822182**	10	64995484	REEP3	intron	A	33.8%	4.602 (0.750)	8.7×10^{-10}	0.2 (7)	16452
**rs3847326**	10	65001865	REEP3	intron	A	48.8%	3.199 (0.701)	5.04×10^{-6}	0.02 (48.8)	16452
**rs10761786**	10	65006213	REEP3	intron	T	59.9%	-3.715 (0.720)	2.51×10^{-7}	0.55 (0)	16452
**rs10822186**	10	65020389	REEP3	intron	A	59.8%	-3.741 (0.722)	2.24×10^{-7}	0.47 (0)	16452

**rs10761787**	10	65023761	REEP3	intron	A	57.7%	-3.511 (0.724)	1.23×10^{-6}	0.43 (0)	16452
**rs10897445**	11	63253388	RTN3	intron	T	91.5%	-5.849 (1.269)	4.01×10^{-6}	0.72 (0)	16403
**rs627055**	11	63645869	MACROD1	intron	T	14.1%	4.929 (1.015)	1.2×10^{-6}	0.49 (0)	16412
**rs1123251**	11	63674177	MACROD1	intron	T	9.7%	6.181 (1.223)	4.35×10^{-7}	1 (0)	16407
**rs4980525**	11	63704930			A	14.6%	4.716 (1.027)	4.42×10^{-6}	0.51 (0)	16452
**rs2244625**	11	63782720	PLCB3	coding-synonymous	A	27.3%	4.221 (0.885)	1.87×10^{-6}	0.15 (15.4)	16452
**rs3815362**	11	63790131	PLCB3	intron	T	9.0%	6.209 (1.396)	8.72×10^{-6}	0.58 (0)	16450
**rs1317494**	11	63793264	BAD	near-gene-3	C	92.2%	-6.702 (1.454)	4.01×10^{-6}	0.53 (0)	16452
**rs11231741**	11	63803461	BAD	intron	T	6.5%	7.392 (1.467)	4.65×10^{-7}	0.71 (0)	15495
**rs477895**	11	63805488	BAD	intron	T	54.7%	4.180 (0.768)	4.91×10^{-8}	0.17 (11.4)	16452
**rs2232410**	11	63815398	KCNK4	utr-5	A	42.5%	-5.685 (1.107)	2.81×10^{-7}	0.2 (4.7)	8389
**rs493052**	11	63834956	ESRRA	intron	A	42.3%	-5.877 (1.103)	9.92×10^{-8}	0.17 (9.2)	8391
**rs9787810**	11	63841874	PRDX5	near-gene-5	T	9.2%	6.059 (1.339)	6.04×10^{-6}	0.54 (0)	16452
**rs558864**	11	63863719	CCDC88B	near-gene-5	T	11.7%	-5.082 (1.074)	2.21×10^{-6}	0.77 (0)	16411
**rs2076853**	11	64035213			A	22.1%	3.924 (0.877)	7.61×10^{-6}	0.08 (29.9)	15491
**rs7936185**	11	64062028			T	10.6%	6.326 (1.202)	1.43×10^{-7}	0.58 (0)	16452
**rs4930420**	11	64063633			C	11.1%	6.414 (1.200)	9.16×10^{-8}	0.59 (0)	16452
**rs4930423**	11	64068473			T	89.0%	-5.402 (1.135)	1.94×10^{-6}	0.09 (26.3)	16452
**rs11231816**	11	64068979			A	10.8%	5.431 (1.133)	1.63×10^{-6}	0.09 (26.7)	16414
**rs7124676**	11	64069867			A	12.6%	5.527 (1.086)	3.63×10^{-7}	0.17 (11.7)	16395

**rs4930426**	11	64072490			C	87.0%	-5.411 (1.091)	7.11×10^{-7}	0.17 (12.6)	16452
**rs523200**	11	64289155	SF1	utr-3	A	23.9%	4.186 (0.884)	2.19×10^{-6}	0.49 (0)	16452
**rs680273**	11	64296211	SF1	intron	C	23.8%	4.145 (0.879)	2.4×10^{-6}	0.49 (0)	16415
**rs650506**	11	64453962	PPP2R5B	intron	T	73.2%	-3.943 (0.883)	7.9×10^{-6}	0.16 (14)	16451
**rs615709**	11	64466377	LOC283129	intron	T	28.5%	4.227 (0.852)	7.1×10^{-7}	0.14 (17.8)	16452
**rs11065884**	12	110303084	LOC642580	intron	A	28.8%	-3.681 (0.787)	2.9×10^{-6}	0.97 (0)	16452
**rs10849947**	12	110349067	SH2B3	intron	T	32.9%	-4.214 (0.757)	2.55×10^{-8}	0.77 (0)	16452
**rs11065900**	12	110357059	SH2B3	intron	C	34.6%	4.095 (0.754)	5.72×10^{-8}	0.85 (0)	15450
**rs7299305**	12	110358652	SH2B3	intron	C	65.4%	-4.155 (0.753)	3.42×10^{-8}	0.9 (0)	15483
**rs7296313**	12	110362909	SH2B3	intron	T	45.4%	-3.519 (0.708)	6.73×10^{-7}	0.89 (0)	16452
**rs2238154**	12	110366868	SH2B3	intron	A	66.8%	4.245 (0.749)	1.48×10^{-8}	0.84 (0)	16450
**rs14555**	12	110373699	SH2B3	utr-3	A	65.2%	-4.071 (0.759)	8.32×10^{-8}	0.79 (0)	15271
**rs10849949**	12	110377920	ATXN2	intron	A	33.2%	-4.177 (0.750)	2.55×10^{-8}	0.88 (0)	16385
**rs2301622**	12	110379586	ATXN2	intron	C	32.8%	-4.218 (0.769)	4.11×10^{-8}	0.74 (0)	15525
**rs11065915**	12	110390377	ATXN2	intron	A	65.9%	-4.104 (0.735)	2.36×10^{-8}	0.96 (0)	16412
**rs10161383**	12	110400664	ATXN2	intron	T	34.1%	4.065 (0.735)	3.27×10^{-8}	0.96 (0)	16408
**rs1029388**	12	110411284	ATXN2	intron	T	32.7%	-4.057 (0.756)	8.18×10^{-8}	0.74 (0)	16392
**rs11065932**	12	110424428	ATXN2	intron	A	66.0%	-4.062 (0.736)	3.41×10^{-8}	0.97 (0)	16404
**rs6490162**	12	110425503	ATXN2	intron	T	44.6%	-3.326 (0.707)	2.54×10^{-6}	0.9 (0)	16451
**rs628825**	12	110436233	ATXN2	intron	T	66.4%	4.243 (0.750)	1.57×10^{-8}	0.85 (0)	16452

**rs630512**	12	110436550	ATXN2	intron	T	66.4%	4.257 (0.751)	1.43×10^{-8}	0.84 (0)	16451
**rs16941541**	12	110438087	ATXN2	intron	A	8.7%	-5.837 (1.261)	3.67×10^{-6}	0.66 (0)	16452
**rs11065936**	12	110441414	ATXN2	intron	T	33.6%	4.007 (0.761)	1.42×10^{-7}	0.94 (0)	15418
**rs657197**	12	110450041	ATXN2	intron	A	66.2%	4.294 (0.750)	1.03×10^{-8}	0.89 (0)	16451
**rs607316**	12	110453831	ATXN2	intron	T	55.2%	3.280 (0.705)	3.33×10^{-6}	0.9 (0)	16452
**rs7978331**	12	110455616	ATXN2	intron	A	65.9%	-4.077 (0.735)	2.93×10^{-8}	0.96 (0)	16413
**rs7136679**	12	110456662	ATXN2	intron	T	65.9%	-4.074 (0.735)	2.95×10^{-8}	0.96 (0)	16414
**rs7979403**	12	110462527	ATXN2	intron	C	66.3%	-4.100 (0.821)	5.85×10^{-7}	0.93 (0)	12816
**rs41500449**	12	110469184	ATXN2	intron	T	33.7%	4.053 (0.802)	4.3×10^{-7}	0.84 (0)	13733
**rs11065951**	12	110479861	ATXN2	intron	T	33.5%	3.947 (0.739)	9.43×10^{-8}	0.97 (0)	16398
**rs7308857**	12	110483583	ATXN2	intron	T	65.4%	-4.092 (0.744)	3.85×10^{-8}	0.98 (0)	16452
**rs616559**	12	110487733	ATXN2	intron	T	45.2%	-3.265 (0.703)	3.45×10^{-6}	0.92 (0)	16415
**rs616513**	12	110487766	ATXN2	intron	T	66.1%	4.191 (0.750)	2.3×10^{-8}	0.85 (0)	16451
**rs16941578**	12	110493082	ATXN2	intron	T	33.6%	3.987 (0.736)	6.01×10^{-8}	0.98 (0)	16408
**rs7972340**	12	110493797	ATXN2	intron	C	33.6%	4.093 (0.757)	6.42×10^{-8}	0.96 (0)	15473
**rs12369009**	12	110504182	ATXN2	intron	T	33.8%	-4.202 (0.750)	2.14×10^{-8}	0.84 (0)	16452
**rs695871**	12	110521383	ATXN2	intron	C	32.4%	-4.186 (0.769)	5.17×10^{-8}	0.8 (0)	16452
**rs12308557**	12	110522500			C	65.6%	-4.172 (0.754)	3.18×10^{-8}	0.96 (0)	15448
**rs9300319**	12	110549280			T	33.3%	-4.262 (0.752)	1.42×10^{-8}	0.71 (0)	16452
**rs10744773**	12	110571193	BRAP	intron	A	66.7%	4.034 (0.755)	9.12×10^{-8}	0.64 (0)	16452

**rs3742001**	12	110587531	BRAP	intron	T	66.3%	4.347 (0.749)	6.58 x 10^{-9}	0.78 (0)	16452
**rs7136874**	12	110598041	BRAP	intron	T	66.5%	4.375 (0.749)	5.23 x 10^{-9}	0.79 (0)	16413
**rs6490294**	12	110674821	ACAD10	intron	A	66.3%	4.384 (0.749)	4.78 x 10^{-9}	0.71 (0)	16375
**rs886205**	12	110688810	ALDH2	utr-5	A	33.9%	-4.373 (0.749)	5.28 x 10^{-9}	0.69 (0)	16452
**rs16941669**	12	110730020	ALDH2	intron	T	90.9%	5.886 (1.237)	1.97 x 10^{-6}	0.27 (0)	16452
**rs7296651**	12	110731337	ALDH2	intron	C	33.4%	-4.198 (0.761)	3.42 x 10^{-8}	0.49 (0)	16409
**rs2106697**	12	110744448			T	33.8%	-4.271 (0.751)	1.27 x 10^{-8}	0.51 (0)	16452
**rs6489818**	12	110794963	MAPKAPK5	intron	A	66.2%	4.256 (0.752)	1.51 x 10^{-8}	0.63 (0)	16451
**rs3742000**	12	110822922			T	33.5%	-4.100 (0.751)	4.82 x 10^{-8}	0.62 (0)	16375
**rs11066099**	12	110876419	TMEM116	intron	C	33.1%	-3.874 (0.751)	2.53 x 10^{-7}	0.45 (0)	16452
**rs9630276**	12	110889221	TMEM116	intron	A	66.6%	4.085 (0.752)	5.57 x 10^{-8}	0.62 (0)	16451
**rs4767264**	12	110910129	TMEM116	intron	T	33.2%	-4.154 (0.764)	5.5 x 10^{-8}	0.64 (0)	16303
**rs7954482**	12	110920399	TMEM116	intron	A	32.1%	3.498 (0.747)	2.81 x 10^{-6}	0.95 (0)	16414
**rs11066121**	12	110921060	TMEM116	intron	C	31.8%	3.486 (0.749)	3.27 x 10^{-6}	0.94 (0)	16452
**rs16941804**	12	110925381	TMEM116	intron	T	73.0%	-3.772 (0.788)	1.69 x 10^{-6}	0.87 (0)	16443
**rs2339941**	12	110975147	C12orf30	intron	T	66.1%	3.833 (0.751)	3.28 x 10^{-7}	0.76 (0)	16450
**rs7972112**	12	110989565	C12orf30	intron	A	65.5%	3.657 (0.801)	5.01 x 10^{-6}	0.2 (0.7)	14697
**rs10850003**	12	111055552	TRAFD1	intron	A	66.3%	3.697 (0.750)	8.34 x 10^{-7}	0.76 (0)	16413
**rs7297415**	12	111145487	C12orf51	intron	A	8.8%	-5.847 (1.237)	2.27 x 10^{-6}	0.44 (0)	16452
**rs8109288**	19	16046559	TPM4	near-gene-5	A	9.7%	-8.724 (1.403)	5.02 x 10^{-10}	0.35 (0)	16452

**rs6070696**	20	57031040	TUBB1	intron	A	82.9%	-4.699 (0.912)	2.55 x 10^{-7}	0.33 (0)	16396
**rs151358**	20	57043454	SLMO2	utr-3	A	17.4%	4.706 (0.929)	4.02 x 10^{-7}	0.54 (0)	16452
**rs151361**	20	57047397	SLMO2	intron	A	74.3%	-4.495 (0.783)	9.44 x 10^{-9}	0.04 (40.1)	16393

I-square = a measure of heterogeneity among studies that cannot be explained by chance; Het-P = Cochrane Q p-value to assess heterogeneity
