# Supplementary material for: A Meta-Analysis and Genome-Wide Association Study of Platelet Count and Mean Platelet Volume in African Americans
Source: PLoS Genet. 2012 Mar 8;8(3):e1002491. doi: 10.1371/journal.pgen.1002491 (PMC3299192; doi:10.1371/journal.pgen.1002491)
Supplement: Table S3 — List of all SNPs that were significant at p-value<10−6 in regions with at least one SNP with GWAS threshold in mean platelet volume meta-analysis. (PDF) [file pgen.1002491.s007.pdf]

**Table S3:** List of all SNPs that were significant at p-value < $10^{-6}$ in regions with at least one SNP with GWAS threshold in mean platelet volume meta-analysis

| SNP | Chr | Position | Gene | Role | Coded Allele | Allele Frequency | Effect Size (SE) | P Value | Het-P (I-square) | N |
|---|---|---|---|---|---|---|---|---|---|---|
| **rs4727659** | 7 | 106144723 | | | T | 23.4% | 0.122 (0.026) | $2.84 \times 10^{-6}$ | 0.20 (0) | 3901 |
| **rs342293** | 7 | 106159455 | | | G | 61.5% | -0.155 (0.024) | $5.84 \times 10^{-11}$ | 0.30 (0) | 3904 |
| **Rs342294** | 7 | 106159858 | | | T | 61.4% | -0.162 (0.033) | $7.37 \times 10^{-7}$ | 0.99 (0) | 1989 |
| **rs342295** | 7 | 106159996 | | | C | 37.6% | 0.159 (0.024) | $3.68 \times 10^{-11}$ | 0.20 (0.4) | 3905 |
| **rs342296** | 7 | 106160139 | | | G | 37.2% | 0.161 (0.024) | $1.44 \times 10^{-11}$ | 0.25 (0) | 3904 |
| **rs342298** | 7 | 106160882 | | | C | 37.3% | 0.161 (0.024) | $3.61 \times 10^{-11}$ | 0.33 (0) | 3905 |
| **rs11653144** | 17 | 24699352 | Near TAOK1 | | C | 55.8% | 0.132 (0.024) | $4.17 \times 10^{-8}$ | 0.48 (0) | 3905 |
| **rs7213208** | 17 | 24756995 | | | G | 47.0% | -0.128 (0.024) | $1.30 \times 10^{-7}$ | 0.29 (0) | 3905 |
| **rs7208363** | 17 | 24757534 | | | T | 45.9% | -0.131 (0.026) | $5.31 \times 10^{-7}$ | 0.18 (3.2) | 3905 |
| **rs11655000** | 17 | 24791469 | | | C | 33.0% | -0.121 (0.027) | $6.33 \times 10^{-6}$ | 0.44 (0) | 3905 |
| **rs9900280** | 17 | 24793724 | | | G | 64.3% | 0.122 (0.025) | $1.37 \times 10^{-6}$ | 0.53 (0) | 3905 |
| **rs12943131** | 17 | 24887832 | | | G | 64.4% | 0.124 (0.025) | $1.04 \times 10^{-6}$ | 0.52 (0) | 3905 |
| **rs8109288** | 19 | 16046559 | TPM4 | near gene-5’ | G | 8.4% | 0.261 (0.044) | $3.30 \times 10^{-9}$ | 0.84 (0) | 3905 |

I-square = a measure of heterogeneity among studies that cannot be explained by chance; Het-P = Cochrane Q p-value to assess heterogeneity
