# Supplementary material for: A Meta-Analysis and Genome-Wide Association Study of Platelet Count and Mean Platelet Volume in African Americans
Source: PLoS Genet. 2012 Mar 8;8(3):e1002491. doi: 10.1371/journal.pgen.1002491 (PMC3299192; doi:10.1371/journal.pgen.1002491)
Supplement: Table S4 — Individual study results for the top significant SNP from each region. (PDF) [file pgen.1002491.s008.pdf]

Table S4: Individual study results for the top significant SNP from each region

SNP[a]	ARIC		CARDIA		GeneSTAR		HANDLS		Health ABC		JHS		WHI	
	**ES (SE)**	**P-value**	**ES (SE)**	**P-value**	**ES (SE)**	**P-value**	**ES (SE)**	**P-value**	**ES (SE)**	**P-value**	**ES (SE)**	**P-value**	**ES (SE)**	**P-value**
Platelet Count														
**rs12526480 G/T**	-4.047 (1.934)	0.036	-5.770 (3.380)	0.088	-8.455 (3.329)	0.011	-8.901 (3.550)	0.012	-4.962 (2.994)	0.098	-2.200 (2.082)	0.291	-3.982 (1.041)	1.31×10^{-4}
**rs210134 A/G**	-5.842 (2.019)	3.8×10^{-3}	-2.520 (3.500)	0.472	-11.360 (3.437)	9.50×10^{-4}	-9.971 (3.568)	5.20×10^{-3}	0.261 (3.101)	0.933	-7.171 (2.096)	6.34×10^{-4}	-6.282 (1.050)	2.26×10^{-9}
**rs9494145 C/T**	9.768 (3.826)	0.011	6.730 (6.090)	0.270	11.159 (6.161)	0.070	7.553 (6.640)	0.255	8.587 (5.081)	0.091	9.916 (3.951)	0.012	7.353 (1.819)	5.29×10^{-5}
**rs13236689 G/T**	3.564 (1.821)	0.050	7.030 (3.100)	0.024	6.194 (3.009)	0.040	5.694 (3.193)	0.075	0.207 (2.835)	0.942	4.514 (1.920)	0.019	4.122 (0.956)	1.61×10^{-5}
**rs342293 G/C**	-1.879 (1.831)	0.305	-6.897 (3.170)	0.030	-2.485 (3.462)	0.473	-10.425 (3.352)	1.87×10^{-3}	-7.227 (2.984)	0.016	-1.446 (1.927)	0.453	-4.231 (0.964)	1.15×10^{-5}
**rs7896518 G/A**	5.959 (1.875)	1.5×10^{-3}	12.60 (3.230)	1.03×10^{-4}	9.535 (3.365)	4.60×10^{-3}	5.898 (3.355)	0.079	1.638 (3.100)	0.587	3.343 (2.004)	0.096	4.587 (1.001)	4.57×10^{-6}
**rs477895**	-6.325	2.1×10^{-3}	3.790	0.269	-4.630	0.148	-7.811	0.012	-5.967	0.040	-5.284	0.011	-3.408	1.29×10^{-3}

**C/T**	(2.058)		(3.420)		(3.197)		(3.097)		(2.907)		(2.071)		(1.059)	
**rs6490294** **C/A**	-4.679 (1.911)	0.014	-4.307 (3.381)	0.203	-5.880 (3.239)	0.069	0.235 (3.432)	0.945	-3.563 (3.116)	0.253	-7.087 (2.066)	6.14×10^{-4}	-4.068 (1.01)	5.70×10^{-5}
**rs8109288** **A/G**	-8.166 (3.695)	0.027	-7.060 (6.990)	0.312	-10.111 (5.280)	0.056	-19.783 (5.614)	4.25×10^{-4}	-4.955 (4.843)	0.306	-13.62 (4.038)	7.58×10^{-4}	-7.013 (1.948)	3.16×10^{-4}
**rs151361** **G/A**	5.233 (2.014)	0.0094	5.496 (3.490)	0.116	14.107 (3.294)	1.85×10^{-5}	0.224 (3.630)	0.951	0.685 (3.249)	0.833	1.209 (2.117)	0.568	4.763 (1.062)	7.34×10^{-6}
Mean Platelet Volume														
**rs342296** **A/G**	0.079 (0.058)	0.176			0.207 (0.058)	6.18×10^{-6}	0.287 (0.095)	0.0024	0.021 (0.171)	0.903	0.153 (0.032)	1.50×10^{-6}		
**rs11653144** **C/T**	-0.148 (0.059)	0.012			-0.090 (0.046)	0.049	-0.106 (0.094)	0.256	-0.104 (0.179)	0.562	-0.163 (0.032)	5.50×10^{-7}		
**rs8109288** **A/G**	0.246 (0.093)	0.035			0.243 (0.074)	0.0011	0.359 (0.159)	0.024	-0.001 (0.274)	0.998	0.278 (0.064)	1.3×10^{-5}		

[a] minor allele always listed first; minor allele = coded/effect allele; ES = effect size
