# Supplementary material for: A Meta-Analysis and Genome-Wide Association Study of Platelet Count and Mean Platelet Volume in African Americans
Source: PLoS Genet. 2012 Mar 8;8(3):e1002491. doi: 10.1371/journal.pgen.1002491 (PMC3299192; doi:10.1371/journal.pgen.1002491)
Supplement: Table S5 — Association analysis of rs12526480 SNP with selected phenotypes. (PDF) [file pgen.1002491.s009.pdf]

Table S5: Association analysis of rs12526480 SNP with selected phenotypes

Phenotype	Beta	SE	P-value	N
**Hemoglobin (g/dL)**	0.0078	0.0134	0.56	16480
**Hematocrit (%)**	0.0285	0.0393	0.47	16491
**MCV (fL)**	-0.0331	0.0501	0.51	6422
**RBC (million/µL)**	0.0085	0.0109	0.43	4802
**Ferritin (ng/mL)**	0.0154	0.0238	0.52	2798
**Uric acid (mg/dL)**	0.0275	0.0505	0.59	936
