# Supplementary material for: A Meta-Analysis and Genome-Wide Association Study of Platelet Count and Mean Platelet Volume in African Americans
Source: PLoS Genet. 2012 Mar 8;8(3):e1002491. doi: 10.1371/journal.pgen.1002491 (PMC3299192; doi:10.1371/journal.pgen.1002491)
Supplement: Table S6 — Conditional analysis of the significant SNPs in at the 11q13 locus for platelet count. (PDF) [file pgen.1002491.s010.pdf]

Table S6: Conditional analysis of the significant SNPs in at the 11q13 locus for platelet count

Gene	SNP	Beta	SE	P	Beta	SE	P	Beta	SE	P	Beta	SE	P
		no adjustment			adjusted for rs477895			adjusted for rs4930420			adjusted for rs2244625		
**BAD**	rs477895	3.456	1.093	0.002				3.024	1.652	0.006	2.214	1.282	0.084
**SLC22A11**	rs4930420	4.719	1.628	0.004	3.969	1.652	0.016				3.598	1.685	0.033
**PLCB3**	rs2244625	4.000	1.226	0.001	2.707	1.439	0.070	3.305	1.685	0.009			
