# Supplementary material for: A Meta-Analysis and Genome-Wide Association Study of Platelet Count and Mean Platelet Volume in African Americans
Source: PLoS Genet. 2012 Mar 8;8(3):e1002491. doi: 10.1371/journal.pgen.1002491 (PMC3299192; doi:10.1371/journal.pgen.1002491)
Supplement: Table S7 — Association of loci previously reported with platelet count in Caucasians, Japanese, or African American populations (from references 13, 15, and 16). (PDF) [file pgen.1002491.s011.pdf]

Table S7: Association of loci previously reported with platelet count in Caucasians, Japanese, or African American populations (from references 13, 15, and 16)

Gene	Platelet count associations reported in literature					Platelet count associations in current study		
	Population (N)	SNP (chr:position)	allele (MAF)	ES (SE)	P-value	Allele (MAF)	ES(SE)	P-value
**GP1BA**	J (14806)	rs6065 (17:4777161)	T (0.12)	0.124 (0.018)	2.13×10^{-17}	T (0.23)	3.649 (0.919)	7.19×10^{-5}
**SH2B3**[a]	J (14806)	rs739496 (12:110372042)	A (0.16)	-0.141 (0.016)	4.75×10^{-19}	G (0.32)	-0.028 (0.740)	0.97
**ARHGEF3**[b]	W (13943)	rs12485738 (3:56840816)	A (0.36)	-5.164 (0.689)	6.6×10^{-14}	A (0.34)	-0.201 (0.736)	0.78
**HBS1L/MYB**	W (13943)	rs9402686 (6:135469510)	A (0.22)	5.503 (0.750)	2.2×10^{-13}	A (0.08)	7.236 (1.312)	3.51×10^{-8}
	J (14806)	rs7775698 (6:135460328)	T (0.34)	0.093 (0.012)	2.54×10^{-14}	T (0.21)	4.008 (0.901)	8.68×10^{-6}
**PIK3CG**	W (13943)	rs342293 (7:106159455)	G (0.45)	-3.684 (0.668)	3.5×10^{-8}	G (0.39)	-4.055 (0.717)	1.58×10^{-8}
**AK3-RCL1**	W (13943)	rs385893 (9:4753176)	T (0.44)	6.264 (0.753)	8.5×10^{-17}	C (0.29)	2.518 (0.968)	9.3×10^{-3}
	J (14806)		T (0.25)	-0.099 (0.014)	2.95×10^{-13}			
**THPO**[c]	J (14806)	rs6141 (3:185572960)	T (0.45)	0.076 (0.012)	5.38×10^{-11}	C (0.10)	-1.996 (1.228)	0.10
**BAK1**	W (13943)	rs210135 (6:33648670)	T (0.32)	5.438 (0.868)	3.7×10^{-10}	T (0.28)	-5.988 (0.784)	2.18×10^{-14}
**BAK1**	AA (7112)	rs449242 (6:33604692)	T (0.33)	-0.126 (0.023)	6.2×10^{-8}	T (0.37)	-4.408 (0.737)	2.26×10^{-9}
**BET1L**	W (13943)	rs11602954 (11:192856)	A (0.23)	6.116 (0.859)	1.1×10^{-12}	A (0.07)	3.122 (1.441)	0.03
**ATXN2**[d]	W (13943)	rs11065987 (12:110556807)	G (0.34)	5.073 (0.692)	2.2×10^{-13}	G (0.07)	2.814 (2.072)	0.17
**PTPN11**	W (13943)	rs11066301 (12:111355755)	G (0.35)	4.650 (0.680)	7.7×10^{-12}	G (0.09)	3.146 (1.418)	0.03
**TPM4**	W (23439)	rs8109288 (19:16046559)	A (0.02)	-0.232 (0.044)	3.0×10^{-7}	A (0.10)	-8.724 (1.403)	5.03×10^{-10}
	AA (7112)		A (0.09)	-0.159 (0.030)	3.0×10^{-7}			
**NFE2-COPZ1**	AA (7112)	rs10876550 (12:52998575)	G (0.12)	-0.135 (0.027)	1.6×10^{-6}	G (0.13)	-3.631 (1.041)	4.84×10^{-4}

MAF = minor allele frequency; ES = effect size; SE = standard error; MPV = mean platelet volume; J = Japanese; W = Caucasians; AA = African Americans

The following SNPs in the current study were close to the previously reported SNPs associated with platelet count and had low p values:

[a] rs14555 is about 1.7 kbps away from rs739496 and has a p-value of 8.32×10^{-8}, minor allele=T, MAF = 35%, effect size (SE) = 4.071(0.759) . In HapMap data, rs14555 is monomorphic in CEU population.

[b] rs17216816 is about 6.7 kbp away from rs12485738 and has a p-value of 4.04×10^{-5}, minor allele = T, MAF = 14%, effect size (SE) = 4.297 (1.047). In HapMap data, the two SNPs (rs17216816 and rs12485738) have r^2 of 87% in CEU population and both SNPs are in the same LD block.

[c] rs10513797 is about 2.2 kbp away from rs6141 and has a p-value of 8.16×10^{-5}, minor allele = A, MAF = 21%, effect size (SE) = 3.349 (0.850). In HapMap data, the two SNPs (rs10513797 and rs6141) have r^2 of 85% in JPT population and are in the same LD block.

[d] rs9300319 is about 7.5 kbp away from rs11065987 and has a p-value of 1.42×10^{-8}, minor allele = T, MAF = 33%, effect size (SE) = -4.26 (0.752). In HapMap data, the two SNPs (rs9300319 and rs11065987) have r^2 of 19% and are in the same LD block.

Minor allele frequencies may be different for platelet count and MPV in the current study due to different number of studies
