# Supplementary material for: A Meta-Analysis and Genome-Wide Association Study of Platelet Count and Mean Platelet Volume in African Americans
Source: PLoS Genet. 2012 Mar 8;8(3):e1002491. doi: 10.1371/journal.pgen.1002491 (PMC3299192; doi:10.1371/journal.pgen.1002491)
Supplement: Table S8 — Association of loci previously reported with mean platelet volume in Caucasians (N = 13943)(from reference [15]). (PDF) [file pgen.1002491.s012.pdf]

Table S8: Association of loci previously reported with mean platelet volume in Caucasians (N=13943)(from reference 15)

Gene	MPV associations reported in literature				Current study MPV		
	SNP (chr:position)	Allele (MAF)	ES(SE)	P-value	Allele (MAF)	ES(SE)	P-value
**WDR66**	rs7961894 (12:120849966)	T (0.12)	0.031 (0.002)	7.24×10^{-44}	T (0.02)	0.375 (0.079)	2.24×10^{-6}
**ARHGEF3**[a]	rs12485738 (3:56840816)	A (0.36)	0.015 (0.001)	5.5×10^{-31}	A (0.34)	-0.020 (0.024)	0.41
**DNM3**[b]	rs10914144 (1:170216373)	T (0.17)	0.013 (0.002)	2.1×10^{-14}	T (0.28)	-0.021 (0.025)	0.394
**TPM1**	rs11071720 (15:61129049)	T (0.37)	0.011 (0.002)	1.9×10^{-8}	C (0.43)	-0.062 (0.024)	0.011
**BET1L**[c]	rs11602954 (11:192856)	A (0.23)	0.013 (0.002)	1.3×10^{-14}	A (0.06)	-0.084 (0.047)	0.078
**TMCC2**	rs1668873 (1:203502613)	A (0.33)	0.012 (0.001)	1.4×10^{-20}	A (0.13)	-0.102 (0.033)	1.93×10^{-3}
**TAOK1**	rs2138852 (17:24727475)	C (0.44)	0.016 (0.002)	1.4×10^{-22}	C (0.16)	-0.089 (0.033)	6.56×10^{-3}
**JMJD1C**[d]	rs2393967 (10:64803162)	C (0.37)	0.014 (0.002)	3.3×10^{-21}	C (0.06)	-0.047 (0.062)	0.42
**PIK3CG**	rs342293 (7:106159455)	G (0.45)	0.015 (0.001)	1.6×10^{-33}	G (0.38)	0.155 (0.024)	5.84×10^{-11}
**SIRPA**[e]	rs6136489 (20:1871734)	G (0.26)	0.010 (0.002)	7.7×10^{-11}	T (0.33)	0.013 (0.020)	0.58
**EHD3**	rs647316 (2:31318333)	A (0.25)	0.010 (0.002)	3.2×10^{-11}	A (0.11)	0.094 (0.041)	0.022
**CD226**	rs893001 (18:65667825)	A (0.47)	0.110 (0.002)	1.4×10^{-10}	C (0.48)	0.091 (0.036)	0.012

MAF = minor allele frequency; ES = effect size; SE = standard error; MPV = mean platelet volume; W = Caucasians

The following SNPs in the current study were close to the previously reported SNPs associated with MPV and had low p values:

a rs13074522 is about 4.9 kbp away from rs12485738 with p=0.028, minor allele =T; MAF: 13%, and effect size (SE) = 0.074(0.034). In HapMap data, the two SNPs (rs13074522 and rs12485738) have r^2 of 72% in CEU population and both SNPs are in the same LD block.

b rs10914127 is about 5.8 kbp away from rs10914144 with p = 0.015, minor allele = A, MAF = 22% and effect size(SE) = -0.067(0.027). In HapMap data, the two SNPs (rs10914127 and rs10914144) have r^2 of 6% in CEU population and both SNPs are in the same LD block.

c rs7934919 is about 22 kbp away from rs11602954 with p = 0.026, minor allele C; MAF = 4% and effect size(SE) = -0.135(0.060). In HapMap data, the two SNPs (rs7934919 and rs11602954) have r^2 of 6% in CEU population and both SNPs are in different LD blocks.

[d] rs7923609 is about 0.7 kbp away from rs2393967 with $p = 9.9 \times 10^{-6}$, minor allele = G, MAF = 22%, and effect size (SE) = 0.106(0.024). In HapMap data, the two SNPs (rs7923609 and rs2393967) have r^2 of 60% in CEU population and both SNPs are in the same LD block.

[e] rs3828016 is about 6.4 kbp away from rs6136489 with p=0.013, minor allele = A, MAF = 20%, and effect size (SE) = -0.072(0.029).). In HapMap data, the two SNPs (rs3828016 and rs6136489) have r^2 of 8.7% in CEU population and both SNPs are in different LD blocks.

Minor allele frequencies may be different for platelet count and MPV in the current study due to different number of studies

All these SNPs were reported in Soranzo et al[15] (N=13943)
