# Supplementary material for: A Meta-Analysis and Genome-Wide Association Study of Platelet Count and Mean Platelet Volume in African Americans
Source: PLoS Genet. 2012 Mar 8;8(3):e1002491. doi: 10.1371/journal.pgen.1002491 (PMC3299192; doi:10.1371/journal.pgen.1002491)
Supplement: Table S9 — Percentage variance in phenotype explained by each SNP for each study. (PDF) [file pgen.1002491.s013.pdf]

Table S9: Percentage variance in phenotype explained by each SNP for each study

SNP	ARIC	CARDIA	GeneSTAR	HANDLS	Health ABC	JHS	WHI
PLATELET COUNT							
**rs12526480**	0.16%	0.28%	0.68%	0.57%	0.20%	0.05%	0.19%
**rs210134**	0.32%	0.05%	1.17%	0.69%	0.00%	0.50%	0.45%
**rs9494145**	0.30%	0.12%	0.38%	0.13%	0.19%	0.32%	0.20%
**rs13236689**	0.14%	0.48%	0.42%	0.27%	0.00%	0.24%	0.23%
**rs342293**	0.04%	0.44%	0.07%	0.87%	0.48%	0.02%	0.23%
**rs7896518**	0.36%	1.36%	0.89%	0.26%	0.02%	0.12%	0.26%
**rs477895**	0.46%	0.14%	0.24%	0.51%	0.34%	0.33%	0.16%
**rs6490294**	0.23%	0.16%	0.34%	0.00%	0.11%	0.54%	0.20%
**rs8109288**	0.27%	0.17%	0.40%	1.16%	0.08%	0.78%	0.24%
**rs151361**	0.24%	0.23%	1.69%	0.00%	0.00%	0.01%	0.24%
MEAN PLATELET VOLUME							
**rs342296**	0.36%	-	2.47%	3.85%	0.01%	1.35%	-
**rs11653144**	1.33%	-	0.49%	0.55%	0.21%	1.62%	-
**rs8109288**	1.15%	-	1.12%	1.98%	0.00%	1.47%	-
