# Supplementary material for: A Meta-Analysis and Genome-Wide Association Study of Platelet Count and Mean Platelet Volume in African Americans
Source: PLoS Genet. 2012 Mar 8;8(3):e1002491. doi: 10.1371/journal.pgen.1002491 (PMC3299192; doi:10.1371/journal.pgen.1002491)
Supplement: Text S1 — Supporting Methods. (DOCX) [file pgen.1002491.s014.docx]

A Meta-analysis and Genome-wide Association Study of Platelet Count and Mean Platelet Volume in African Americans

**Supporting Methods Text**

*Author list:* Rehan Qayyum^1,^*, Beverly M. Snively^2^, Elad Ziv^3^, Michael A. Nalls^4^, Yongmei Liu^5^, Weihong Tang^6^, Lisa R. Yanek^1^, Leslie Lange^7^, Michele Evans^8^, Santhi Ganesh^9^, Melissa A. Austin^10^, Guillaume Lettre^11^, Diane M. Becker^1^, Alan B. Zonderman^12^, Andrew B. Singleton^4^, Tamara B. Harris^13^, Emile R. Mohler^14^, Benjamin A. Logsdon^15^, Charles Kooperberg^15^, Aaron R. Folsom^6^, James G. Wilson^16^, Lewis C. Becker^1, 18^, Alexander P. Reiner^17, 18^

1. GeneSTAR Research Program, Division of General Internal Medicine, Johns Hopkins School of Medicine, Baltimore, MD 21287, USA

2. Department of Biostatistical Sciences, Wake Forest School of Medicine, Winston-Salem, NC 27157, USA

3. Department of Medicine, University of California, San Francisco, CA 94143, USA

4. Laboratory of Neurogenetics, National Institute on Aging, National Institutes of Health, Bethesda, MD 20892, USA

5. Department of Epidemiology and Prevention, Division of Public Health Sciences, Wake Forest University School of Medicine, Winston-Salem, NC 27157, USA

6. Division of Epidemiology and Community Health, University of Minnesota School of Public Health, Minneapolis, MN 55454, USA

7. Department of Genetics, University of North Carolina School of Medicine at Chapel Hill, Chapel Hill, North Carolina 27514, USA

8. Health Disparities Research Section, Clinical Research Branch, National Institute on Aging, National Institutes of Health, Baltimore, Maryland 21225, USA

9. Division of Cardiology, University of Michigan Health System, Ann Arbor, Michigan 48109, USA

10. Department of Epidemiology, University of Washington; Division of Public Health Sciences, Fred Hutchinson Cancer Research Center, Seattle, WA 98195, USA

11. Montreal Heart Institute, Montréal, Québec, H1T 1C8, Canada

12. Laboratory of Personality and Cognition, National Institute on Aging, National Institutes of Health, Baltimore, Maryland 21224, USA

13. Laboratory for Epidemiology, Demography, and Biometry, National Institute on Aging, National Institutes of Health, Baltimore, Maryland, USA

14. Department of Medicine, University of Pennsylvania School of Medicine, Philadelphia, PA 19104, USA

15. Program in Biostatistics and Biomathematics, Division of Public Health Sciences Fred Hutchinson Cancer Research Center Seattle, WA 98109, USA

16. Department of Medicine, University of Mississippi Medical Center, Jackson, MS 39216, USA

17. University of Washington, Department of Epidemiology, Seattle, WA 98195, USA

18. Senior co-authors

* Correspondence: [rqayyum@jhmi.edu](mailto:rqayyum@jhmi.edu)

***Correspondence to:***

Rehan Qayyum, MD

Park 307-A

600 North Wolfe Street

Baltimore, MD 21287

rqayyum@jhmi.edu

1. **DESCRIPTION OF THE DEMOGRAPHICS OF COHORTS INCLUDED IN THE STUDY**

***1. Women's Health Initiative (WHI)-SHARe:*** WHI is one of the largest (n=161,808) studies of women's health ever undertaken in the U.S. There are two major components of WHI: (1) a Clinical Trial (CT) that enrolled and randomized 68,132 women ages 50 – 79 into at least one of three placebo-control clinical trials (hormone therapy, dietary modification, and calcium/vitamin D); and (2) an Observational Study (OS) that enrolled 93,676 women of the same age range into a parallel prospective cohort study.^1^ A diverse population including 26,045 (17%) women from minority groups were recruited from 1993-1998 at 40 clinical centers across the U.S. Of the CT and OS minority participants enrolled in WHI, 12,157 (including 8,515 self identified African American and 3,642 self identified Hispanic subjects) who had consented to genetic research were eligible for the WHI SHARe GWAS project. Budget constraints led to a random selection of 12,157 women; 8,515 (70.1%) were African American and 3642 (66.6%) were Hispanic.  If a sample was selected and DNA was not available, another sample was randomly selected. DNA was extracted by the Specimen Processing Laboratory at the Fred Hutchinson Cancer research Center (FHCRC) using specimens that were collected at the time of enrollment in to the study (between 1993 and 1998). Phlebotomy was performed by trained and certified staff using universal precautions at each of 40 WHI Clinical Centers in 2 mL lavender EDTA tube. Blood was drawn in the fasting state after a 12 hour fast in the seated position. Women were instructed to take all regular medications except for insulin or oral medication used to control diabetes. They were also instructed to take no aspirin or non-steroidal anti-inflammatory drugs (NSAIDs) for 48 hours before the visit. NSAIDs that were taken regularly were continued and taken within the 48 hours before the blood draw, consistent with the participant's usual schedule. They were instructed not to smoke for at least one hour before the blood draw and to perform no vigorous physical activity (such as jogging or bicycling) for at least 8 hours before the blood draw. Platelet counts were performed on fresh specimens at 40 different local laboratories using automated cell counters. [Williams WJ Schneider AS. Examination of the peripheral blood. In: Williams WJ, Beutler E, Erslev AJ. Rundles RW. Eds. Hematology. New York: McGraw-Hill; 1972: 10-22.]

***2. Atherosclerosis Risk in Communities Study (ARIC):*** The ARIC study is a prospective population-based study of atherosclerosis and cardiovascular diseases in 15,792 men and women, including 11,478 whites and 4,314 African Americans, drawn from 4 U.S. communities (suburban Minneapolis, Minnesota; Washington County, Maryland; Forsyth County, North Carolina, and Jackson, Mississippi).^2^ Only self-reported African-American participants were included in this analysis. Participants were between age 45 and 64 years at their baseline examination in 1987-1989. Blood for platelet count analysis was drawn at the baseline exam. Blood for DNA extraction was drawn and participants consented to genetic testing on following visits, primarily visit 2 (1990-1992). After taking into account availability of adequate amounts of high quality DNA, appropriate informed consent and genotyping quality control and assurance procedures, genotype data were available on 2,989 African-American individuals.

***3. Coronary Artery Risk Development in Young Adults (CARDIA):*** The CARDIA study is a prospective, multi-center investigation of the natural history and etiology of cardiovascular disease in African Americans and whites 18-30 years of age at the time of initial examination.^3^ The CARDIA sample was recruited at random during 1985-86 primarily from geographically based populations in Birmingham AL, Chicago IL, and Minneapolis MN and, in Oakland, CA, from the membership of the Kaiser-Permanente Health Plan. The initial examination included 5,115 participants selectively recruited to represent proportionate racial, gender, age, and education groups from four communities: Birmingham, AL; Chicago, IL; Minneapolis, MN; and Oakland, CA. Each participant’s age, race, and sex were self-reported during the recruitment phase and verified during the baseline clinic visit. Details of the study design and procedures for data collection have been published.^3^ From the time of initiation of the study in 1985-1986 (baseline examination), five follow-up examinations have been conducted at years 2, 5, 7, 10, 15, and 20. DNA extraction for genetic studies was performed at the year 10 examination. Blood for platelet analysis was drawn at the baseline exam (between 1985 and 1986). Blood was drawn in the seated position after a 12-hour fast. Platelet counts were performed on fresh specimens at local laboratories using automated cell counters. To test the reliability of the measurements, five specimens at each center were split into three samples and measured at each laboratory. Within-sample variance was 4% of total variance, indicating excellent reproducibility. After taking into account availability of adequate amounts of high quality DNA, appropriate informed consent and genotyping quality control and assurance procedures, genotype data were available on 955 African-American individuals.

**4. *Jackson Heart Study (JHS):*** The Jackson Heart Study (JHS) is a prospective population-based study to seek the causes of the high prevalence of common complex diseases among African Americans in the Jackson, Mississippi metropolitan area, including cardiovascular disease, type-2 diabetes, obesity, chronic kidney disease, and stroke.^4^ During the baseline examination period (2000-2004) 5,301 self-identified African Americans were recruited from four sources, including (1) randomly sampled households from a commercial listing; (2) ARIC participants; (3) a structured volunteer sample that was designed to mirror the eligible population; and (4) a nested family cohort. Unrelated participants were between 35 and 84 years old, and members of the family cohort were ≥ 21 years old when consent for genetic testing was obtained and blood was drawn for DNA extraction. Blood for platelet count was drawn at baseline in the morning after an overnight fast. Based on DNA availability, appropriate informed consent, and genotyping results that met quality control procedures, genotype data were available for 3,030 individuals, including 885 who are also ARIC participants. In the current study, JHS participants who were also enrolled in the ARIC study were analyzed with the ARIC dataset – for this reason, the JHS dataset analyzed here had 2,145 individuals.

***5. Health, Aging, and Body Composition (Health ABC) Study:*** The Health ABC study is a prospective cohort study investigating the associations between body composition, weight-related health conditions, and incident functional limitation in older adults. Health ABC enrolled well-functioning, community-dwelling African-American (n=1,281) and white (n=1,794) men and women aged 70-79 years between April 1997 and June 1998. Participants were recruited from a random sample of all Medicare eligible residents in the Pittsburgh, PA, and Memphis, TN, metropolitan areas. Eligibility requirements included no difficulty with activities of daily living, walking a quarter of a mile, or climbing 10 steps without resting. Participants have undergone annual exams and semi-annual phone interviews. Blood for platelet analysis and genotyping was drawn at the baseline examination***.***

***6. The Healthy Aging in Neighborhoods of Diversity Across the Life Span Study (HANDLS):*** HANDLS is an interdisciplinary, community-based, prospective longitudinal epidemiologic study examining the influences of race and socioeconomic status (SES) on the development of age-related health disparities among socioeconomically diverse African Americans and whites in Baltimore. The study domains include: nutrition, cognition, biologic biomarkers, body composition and bone quality, psychophysiology, physical function and performance, sociodemographics, psychosocial, neighborhood environment and cardiovascular disease. From 2004-2008, a total of 3,722 participants were recruited from Baltimore, MD with mean age 47.7 (range 30-64) years, including 2,200 African Americans (59%) and 1,522 whites (41%); 41% reported household incomes below the 125% poverty delimiter. African Americans subjects with a genome-wide scan (N=863) were included in the current meta-analysis. Following an overnight fast, blood samples for platelet analysis were drawn from the antecubital vein at baseline exam.

***7.* *Genetic Study of Atherosclerosis Risk (GeneSTAR):*** GeneSTAR is a family-based prospective study in which apparently healthy relatives of patients with premature coronary artery disease (age<60 years) were enrolled from Baltimore, Maryland. Hospitalized patients with documented CAD including acute myocardial infarction, coronary artery bypass surgery, percutaneous coronary intervention, angina with angiographic evidence of flow-limiting coronary stenosis, or following sudden cardiac death identified teir healthy relatives. Participating relatives were eligible if they were <60 years of age at the time of enrollment in GeneSTAR and had no known history of CAD and were excluded if they had autoimmune disease, life-threatening co-morbidity or were receiving chronic glucocorticosteroid therapy. The study was approved by the Johns Hopkins Medicine Institutional Review Board and all study participants gave informed consent. For the current study, 934 African-American subjects from 234 families with platelet count and 853 subjects from 228 families with MPV information measured from 2003-2006 underwent GWAS analysis. Platelet function studies were performed on a subset of subjects who had no history of bleeding disorder, were free of serious comorbidities, were not pregnant, and were not taking aspirin or other nonsteroidal anti-inflammatory drugs during the 14 days prior to platelet function tests.

**B. GENOTYPING AND QUALITY CONTROL**

***1. CARe Cohorts:***  ARIC, CARDIA, and JHS samples were genotyped at the Broad Institute using the Affymetrix Genome-Wide Human SNP Array 6.0 (Affy6.0) according to the manufacturer’s recommendations as previously described. This genotyping platform interrogates simultaneously 1.8 million markers for genetic variation (906,600 SNPs and 946,000 copy number variation probes). Genotypes were called using Birdseed v1.33.^5^ Several quality control (QC) procedures were performed on the genotype data, separately for each cohort. Quantity of double stranded DNA was assessed using PicoGreen® (Molecular Probes, Oregon, USA). To confirm sample identity, genotype concordance was evaluated for 24 SNPs genotyped in the same DNA samples using both Sequenom iPLEX and Affy6.0. Genome-wide genotype data was used to estimate identity-by-descent (IBD) between all pairwise combinations of samples in order to identify sample duplicates, contaminated samples, and cryptic relationships.^6^ We also used IBS/IBD measures, as implemented in PREST-Plus^7^, to confirm known pedigree data for JHS. SNPs and samples with an unusually high number of Mendelian errors were excluded. Heterozygosity rates (in the form of inbreeding coefficients) were estimated to identify problematic DNA samples (poor DNA quality or contaminations). DNA samples with a genome-wide genotyping success rate <95%, duplicate discordance or sex mismatch, SNPs with genotyping success rate <90%, monomorphic SNPs, SNPs with minor allele frequency (MAF) <1%, and SNPs that mapped to several genomic locations were removed from the analyses. The Hardy-Weinberg equilibrium (HWE) test was performed for all SNPs, but SNPs were not excluded based uniquely on this criterion given the admixed nature of the cohorts genotyped. After applying all quality control filters, the following numbers of African-American participants were available for analysis: ARIC=2,830, CARDIA=949, and JHS=2,144.

***2. WHI:*** Genotyping was done at Affymetrix Inc on the Affymetrix 6.0 array, using 2 µg DNA at a concentration of 100 ng/uL. 2% (238) additional samples were genotyped as blind duplicates. When needed, multiple attempts were made to genotype samples. Approximately 1% of samples could not be genotyped (n=99). We excluded samples that had call rate below 95% (n=16), that were a duplicate of subjects other than monozygotic twins (n=34), or that had a Y-chromosome (n=1). SNPs that were located on the Y chromosome or were Affymetrix QC probes (not intended for analysis) were also excluded (n=3280). We also flagged SNPs that had call rates below 95% and concordance rates below 98%, leaving us 871,309 unflagged SNPs. We computed IBD coefficients between all pairs of subjects using a random subset of 100,000 SNPs from autosomal chromosomes and using the method of moments approach to a three parameter IBD model using PLINK.^6^ Based on these coefficients we identified pairs of parent-offspring (22 pairs and two trios), monozygotic twins (five pairs) and siblings (192 pairs and five trios). A more thorough confirmatory analysis using a pairwise kinship coefficient estimator^8^ as well as the aforementioned method of moments approach was performed that validated these relationships and identified additional half-siblings. In most analyses we only include one of each pairs of relatives, typically the one with the largest call rate. We were left with 8,421 unique African Americans subjects with an average call rate of 99.8% over the unflagged SNPs. We analyzed 188 pairs of blind duplicate samples. The overall concordance rate was 99.8% (range 94.5-100% over samples, 98.3%-100% over samples with call rate >98%, 98.1-100% over unflagged SNPs).

***3. Health ABC:*** For Health ABC, genotyping was performed by the Center for Inherited Disease Research (CIDR) using the Illumina Human1M-Duo BeadChip system. Genomic DNA was extracted from buffy coat collected during the baseline exam using a PUREGENE® DNA Purification Kit. DNA samples were excluded from the dataset for reasons of sample failure, genotypic sex mismatch, and first-degree relative of an included individual based on genotype data.  In addition, analysis was restricted to SNPs with minor allele frequency > 0.01, SNP call rate ≥ 97%, and HWE p≥10^-6^. SNPs that mapped to multiple locations or where missingness could be predicted from surrounded haplotypes were excluded. Basic genotype quality control and data management were conducted using PLINKv1.06. Genotyping was successful for 1,151,215 SNPs in 1,139 African Americans. For African Americans, genotypes were available on 1,007,948 high quality SNPS for imputation based on a 1:1 mixture of the CEPH:YRI reference panel (release 22, build 36).

***4. HANDLS:*** Genotyping was focused on a subset of participants self-reporting as African American, and was performed at the Laboratory of Neurogenetics, National Institute on Aging, National Institutes of Health. 1024 participants were successfully genotyped to 907,763 SNPS at the equivalent of Illumina 1M SNP coverage (709 samples using Illumina 1M and 1Mduo arrays, the remainder using a combination of 550K, 370K, 510S and 240S to equate the million SNP level of coverage), passing inclusion criteria into the genetic component of the study. Initial inclusion criteria for genetic data in HANDLS includes concordance between self reported sex and sex estimated from X chromosome heterogeneity, > 95% call rate per participant (across all equivalent arrays), concordance between self-reported African ancestry and ancestry confirmed by analyses of genotyped SNPs, and no cryptic relatedness to any other samples at a level of proportional sharing of genotypes > 15% (effectively excluding 1st cousins and closer relatives from the set of participants used in analyses). In addition, SNPs were filtered for HWE p-value > 1 x 10^-7^, missing by haplotype p-values > 1 x 10^-7^, minor allele frequency > 0.01, and call rate > 95%. Basic genotype quality control and data management was conducted using PLINKv1.06. Cryptic relatedness was estimated via pairwise identity by descent analyses in PLINK and confirmed using RELPAIR.

***5. GeneSTAR:*** Genotyping was performed using the Illumina 1Mv1C platform at deCODE Genetics (Reykjavik, Iceland) in 944 self-identified African American participants with platelet count and MPV measurements. We excluded 17 participants with >5% Mendelian errors, gender discrepancies, or ancestry outliers from any of the first 10 principal components from EIGENSTRAT. SNPs that were missing chromosomal location, monomorphic, had minor allele frequency < 0.01, or had a call rate < 90% were excluded from analysis. Of the 1,042,145 successfully genotyped SNPs, 687,132 high quality SNPs were used for imputation.

**C. PRINICIPAL COMPONENT ANALYSIS (PCA) AND ADJUSTMENT FOR POPULATION STRATIFICATION**

PCA was implemented in EIGENSTRAT^9^ on cleaned African-American GWA genotype data using a subset of markers in common between the cohort samples and the reference panels. To identify axes of variation, we included global HapMap and/or HGDP populations (e.g., CEU, YRI, CHB+JPT, Native American) genotyped on the same platform as reference or seed samples. These reference populations were cleaned prior to PCA to remove population outliers. Examination of plots of the main principal components (PCs) for each cohort showed a high degree of correlation of the first PC with global European vs. African ancestry (*r*^2^ >0.90), as calculated independently using the population genetics software STRUCTURE or FRAPPE.^10,11^ Participants were excluded if PC estimates for the first 4 PC were inconsistent with HapMap ASW samples or were PCA outliers (for example, 56 WHI, 8 ARIC, and 2 CARDIA subjects who were self-identified as African American, but who appeared to have less than 10% African ancestry). Study-specific criteria were used to determine the number of principal components used as covariates in regression analyses, ranging from 1 PC to 10 PC.

**D. GENOTYPE IMPUTATION**

Imputation in African-Americans was performed using MACH 1.0.16, which requires phased reference haplotypes (http://www.sph.umich.edu/csg/abecasis/MaCH/). Individuals with pedigree relatedness or cryptic relatedness (pi_hat > 0.05) were filtered prior to imputation, except for GeneSTAR. SNPs with minor allele frequency ≥1%, call rate ≥97% and HWE *P* ≥10^-6^ were used for imputation. A combined CEU+YRI reference panel from HapMap phase 2 (release 22, build 36) was used.^12^ A randomly selected subset of individuals from each cohort sample was used to generate recombination and error rate estimates. These rates were then used to estimate genotype dosages in all sampled individuals across the entire reference panel for over 2 million SNPs. Imputation results were filtered using a minimum imputation quality score, indicated by the RSQ_HAT estimate in MaCH of >0.5 and a minor allele frequency threshold of >1%.

**E. REPLICATION COHORTS**For replication of association findings in African Americans, we used genotyped and imputed SNPs available in additional population-based genome-wide association studies, including 4,324 European American (EA) participants from the Women's Health Initiative (WHI) Genomics and Randomized Trials Network (GARNET) study, 10,585 EA participants from the ARIC study, and 3,462 Hispanic American women from the WHI-SHARe project . WHI-GARNET participants were genotyped using the Ilumina Omni-quad chip at the Broad Institute, and imputation was performed into 1000 Genomes at the GARNET Coordinating Center (University of Washington) using BEAGLE. ARIC and WHI-SHARe participants were genotyped using the Affymetrix 6.0 chip and imputation performed using MaCH. Association testing for typed or imputed SNPs was performed using PLINK.
